# Supplementary material for: Can co-created knowledge mobilisation interventions alter and enhance mindlines to improve childhood eczema care? A UK-based Social Impact Framework evaluation
Source: BMJ Open. 2023 Apr 19;13(4):e065557. doi: 10.1136/bmjopen-2022-065557 (PMC10124191; doi:10.1136/bmjopen-2022-065557)
Supplement: Author's manuscript [file bmjopen-2022-065557.draft_revisions.pdf]

BMJ Open is committed to open peer review. As part of this commitment we make the peer review history of every article we publish publicly available.

When an article is published we post the peer reviewers' comments and the authors' responses online. We also post the versions of the paper that were used during peer review. These are the versions that the peer review comments apply to.

The versions of the paper that follow are the versions that were submitted during the peer review process. They are not the versions of record or the final published versions. They should not be cited or distributed as the published version of this manuscript.

BMJ Open is an open access journal and the full, final, typeset and author-corrected version of record of the manuscript is available on our site with no access controls, subscription charges or pay-per-view fees (http://bmjopen.bmj.com).

If you have any questions on BMJ Open's open peer review process please email info.bmjopen@bmj.com

BMJ Open

Can co-created knowledge mobilisation interventions alter and enhance mindlines to improve childhood eczema care? A Social Impact Framework evaluation.

Journal:	*BMJ Open*
Manuscript ID	bmjopen-2022-065557
Article Type:	Original research
Date Submitted by the Author:	14-Jun-2022

Complete List of Authors:	Cowdell, Fiona; Birmingham City University Faculty of Health Education and Life Sciences, Faculty of Health Education and Life Sciences<br>Lax, Stephanie; University of Nottingham, Centre of Evidence Based Dermatology<br>Van Onselen, Julie; Independent Researcher<br>Pendleton, Rose ; Independent Researcher

Keywords:	Eczema < DERMATOLOGY, QUALITATIVE RESEARCH, Paediatric dermatology < DERMATOLOGY

SCHOLARONE™
Manuscripts

I, the Submitting Author has the right to grant and does grant on behalf of all authors of the Work (as defined in the below author licence), an exclusive licence and/or a non-exclusive licence for contributions from authors who are: i) UK Crown employees; ii) where BMJ has agreed a CC-BY licence shall apply, and/or iii) in accordance with the terms applicable for US Federal Government officers or employees acting as part of their official duties; on a worldwide, perpetual, irrevocable, royalty-free basis to BMJ Publishing Group Ltd ("BMJ") its licensees and where the relevant Journal is co-owned by BMJ to the co-owners of the Journal, to publish the Work in this journal and any other BMJ products and to exploit all rights, as set out in our licence.

The Submitting Author accepts and understands that any supply made under these terms is made by BMJ to the Submitting Author unless you are acting as an employee on behalf of your employer or a postgraduate student of an affiliated institution which is paying any applicable article publishing charge ("APC") for Open Access articles. Where the Submitting Author wishes to make the Work available on an Open Access basis (and intends to pay the relevant APC), the terms of reuse of such Open Access shall be governed by a Creative Commons licence – details of these licences and which Creative Commons licence will apply to this Work are set out in our licence referred to above.

Other than as permitted in any relevant BMJ Author's Self Archiving Policies, I confirm this Work has not been accepted for publication elsewhere, is not being considered for publication elsewhere and does not duplicate material already published. I confirm all authors consent to publication of this Work and authorise the granting of this licence.

For peer review only

Article title: Can co-created knowledge mobilisation interventions alter and enhance mindlines to improve childhood eczema care? A Social Impact Framework evaluation.

Corresponding author information:
Fiona Cowdell
DProf, RN
Professor of Nursing and Health Research
Faculty of Health, Education and Life Sciences
Birmingham City University
220 Ravensbury House
Westbourne Road
Edgbaston
Birmingham
B15 3TN
UK
Telephone: +44 (0)121 300 4345
Email: Fiona.cowdell@bcu.ac.uk
orcid.org/0000-0002-9355-8059

Stephanie Lax
PhD
Research Fellow
Centre of Evidence Based Dermatology
School of Medicine
Applied Health Research Building
University of Nottingham
NG7 2RD
Email: stephanie.lax@nottingham.ac.uk
orcid.org/0000-0002-7000-9364

Julie Van Onselen, RGN, RSCH, DipN, DipM, BA(Hons), Lecturer Practitioner in Dermatology, Dermatology Education Partnership Ltd and Nurse Adviser, National Eczema Society

Rose Pendleton
PPI Parent
c/o Birmingham City University

Word count: 4622

Number of figures: 2

Number of tables: 6

Keywords: atopic eczema, knowledge mobilisation, interventions, mindlines, contributions, social impact

For peer review only - http://bmjopen.bmj.com/site/about/guidelines.xhtml

Abstract

Title: Can co-created knowledge mobilisation interventions alter and enhance mindlines to improve childhood eczema care? A Social Impact Framework evaluation.

Objective: To evaluate the impact of using knowledge mobilisation interventions to alter and enhance mindlines and improve childhood eczema care.

Design: The eczema mindlines study involved three stages: i) mapping and confirming eczema mindlines, ii) intervention development and delivery and iii) analysis of intervention impact. The focus of this paper is on stage three. Data analysis was guided by the Social Impact Framework to address the questions i) what is the impact of this study on individuals and groups? ii) what changes in behaviour and practice have occurred due to their involvement? iii) what mechanisms have enabled these impacts or changes to occur? and iv) what are the recommendations and questions arising from this research?

Settings: A deprived inner-city neighbourhood in central England, and national/international settings.

Participants: Patients, practitioners and wider community members exposed to the interventions locally, nationally and internationally.

Results: Data revealed tangible multi-level, relational and intellectual impacts. Mechanisms that supported impact included: simplicity and consistency of messages adapted to audience, flexibility, opportunism and perseverance, personal interconnectivity and acknowledgment of emotion. Co-created knowledge mobilisation strategies designed to alter and enhance mindlines mediated through knowledge brokering were effective in producing tangible changes in eczema care practice and self-management and in 'mainstreaming' childhood eczema in positive way across communities. These changes cannot be directly attributed to the KMb interventions however, the evidence points to the significant contribution made.

Conclusion: Co-created knowledge mobilisation interventions offer a valuable method of altering and enhancing eczema mindlines across lay-practitioner-wider society boundaries. The Social Impact Framework provides comprehensive method of understanding and documenting the complex web of impact occurring as a result of knowledge mobilisation. This approach is transferable to managing other long-term conditions.

For peer review only - http://bmjopen.bmj.com/site/about/guidelines.xhtml

For peer review only

Strengths and limitations

- First study to assess the impact of interventions to alter and enhance mindlines
- New application of Social Impact Framework in the context of knowledge mobilisation
- Participants from across lay-practitioner-wider society boundaries
- Findings from this evaluation may be transferable to other contexts and conditions

For peer review only

For peer review only - http://bmjopen.bmj.com/site/about/guidelines.xhtml

Can co-created knowledge mobilisation interventions alter and enhance mindlines to improve childhood eczema care? A Social Impact Framework evaluation

Introduction

Co-created knowledge mobilisation (KMb) interventions have the potential to influence the stubborn evidence-practice gap in healthcare but measuring impact of these approaches is challenging. Childhood atopic eczema (AE) is a common and bothersome skin condition (1) which requires regular and ongoing self-management (2). AE is predominantly treated in primary care (3) and a robust evidence base for treatment exists (4, 5). Effective self-management requires a level of shared knowledge, language and understanding between patient and practitioner (6).

Co-methodologies in healthcare are widely considered to be a 'good thing' although the language of 'co' working is not fully defined and remains a fundamentally contested concept (7). The terms co-design, co-production, co-creation, participatory research or participatory design are progressively used, sometimes interchangeably by researchers (8) and research funders (9). Regardless of this, co-methodological working is gaining traction in healthcare (10) and it is widely acknowledged that research engaging end-users is more likely to have an impact on practice (11).

KMb interventions are increasingly used in healthcare to address multiple gaps between evidence, knowledge and action (12). It requires purposeful efforts to create, disseminate and operationalise knowledge from multiple sources (13). KMb is context specific (14), relational (15) and socially constructed (16). It is a rapidly evolving and wide-ranging field; currently there are in excess of 47 models (17) and 71 published reviews (18) and a Google search yields 72,800,000 hits. Selecting approaches to KMb can be problematic with some being highly theoretical and difficult to apply in practice. One pragmatic approach which is firmly embedded in day-to-day practice is alteration and enhancement of 'mindlines'. Mindlines are 'collectively reinforced, internalised tacit guidelines' which underpin clinical decision making (19), particular emphasis is on contextual relevance and application of knowledge. Mindlines are developed from multiple knowledge sources such as communication with colleagues and opinion leaders and from personal tactic knowledge developed over time, knowledge is socially transmitted in the context of its use (19). Mindlines build on the work of Nonaka and colleagues (20) who propose the **S**ocialisation, **E**xternalisation, **C**ombination, **I**nternalisation (SECI) spiral to guide implementation of new knowledge into practice. The SECI spiral comprises, socialisation (surfacing tacit knowledge through shared experiences), externalisation (articulating tacit knowledge into explicit knowledge), combination (combining exposed explicit knowledge with more complex and systematic explicit knowledge, for example clinical guidelines, to

develop new knowledge) and internalisation (embodying this new knowledge as tacit knowledge for day-to-day use).

Impact of KMb is notoriously hard to measure. To date the focus in healthcare has primarily been on moving new knowledge to clinicians and policy makers, with less attention paid to KMb across communities (21, 22). Effective evaluation of KMb activity is essential to better understand if and how stakeholders across communities use new knowledge and to refine strategies (23). The Social Impact Framework, although primarily directed to evaluating co-production offers a comprehensive and structured approach to understanding and documenting micro-meso-macro levels, processes, impacts and mechanisms of the KMb activity and to map the winding pathway of incremental and often subtle changes which are readily overlooked (24). Beckett et al (12) provide a worked example of application of the SIF and their suggested questions are used here to guide analysis (Table 1):

1. What is the impact of this study on individuals and groups? (Outcome 1)
2. What changes in thinking, behaviour and practice have occurred due to their involvement? (Outcome 2)
3. What mechanisms have enabled these impacts or changes to occur? (Outcome 3)
4. What are the recommendations and questions arising from this research? (Outcome 4).

Table 1: Social Impact Review questions (from Beckett et al (12))

Method

The eczema mindlines study involved three phases: **Stage 1:** Mapping and confirming eczema mindlines, **Stage 2:** Intervention development and delivery and **Stage 3:** Analysis of intervention impact (see Figure 1). The focus of this paper is on Phase 3, for context and clarity summaries of Phases 1 & 2 are included.

Insert Figure 1: Evidence sources, stages and outcome measures (adapted from Beckett et al (12))

Phase 1: Mapping and confirming eczema mindlines

Phase 1 comprised two elements. Firstly, an ethnographic study to map lay and practitioner eczema mindlines in one deprived inner-city area in the UK (25, 26). Secondly an interview study with a

For peer review only - http://bmjopen.bmj.com/site/about/guidelines.xhtml

wider population to confirm and expand understanding of lay and practitioner eczema mindlines (27).

Phase 2: Intervention development and delivery

In a series of co-creation workshops involving people living with eczema, practitioners and researchers combined their tacit knowledge and data from phase 1 with existing research evidence. Co-creators concluded that to alter and enhance existing eczema mindlines five key, consistent, evidence-based messages needed to be shared (Table 2).

i)	Eczema is more than just dry skin
ii)	Eczema doesn't just go away
iii)	Moisturisers are for every day
iv)	Steroid creams are okay when you need them
v)	You know your child's eczema best

Table 2: Five co-created eczema messages

Crucially these messages needed to be transmitted across lay-practitioner-wider society boundaries using a range of techniques to enhance shared knowledge, understanding and language. For lay co-creators 'trust' and 'realness' of messages was important and HCPs wanted practical, locally relevant, hints and tips, tailored, 'no faff' approaches (28). KMb interventions were developed in light of the characteristics of evidence and context in order to determine the best approaches (29).

Intervention delivery was grounded in four schools of thought which cumulatively ensured knowledge was mobilised in the right format for the right audience and effectively spread across boundaries as summarised in Table 3.

Knowledge brokering

- Knowledge brokers build networks and facilitate opportunities to share knowledge (30). This was the core of all KMb activity.

'Ba'

- 'Ba' is a shared space for knowledge generation and spreading (31-33) which aligns with the Socialisation, Externalisation, Combination, Internalisation spiral from which mindlines evolved. In this case the Ba space was the locality of the original research, a deprived inner-city catchment. The intention was to achieve a density of KMb in a local

For peer review only - http://bmjopen.bmj.com/site/about/guidelines.xhtml

area to support shared understandings and language across lay-practitioner-wider society boundaries.
Ripple effect model
• Ripple effect was used to amplify the impact of each KMb action, so one event produces effects which spread and produce further effects (34, 35).
Social marketing
• Social marketing goes beyond simply conveying knowledge widely and is intended to directly influence health care actions (36). Here emphasis was on community outreach (37).

Table 3: Underpinning approaches to KMb

Using the strategies outlined above the five co-created messages were shared using multiple interventions as summarised in Table 4. Messages were integrated into a children's book 'The Dragon in My Skin' (hereafter Dragon) with associated animation, song and teacher resources. Dragon resources were endorsed by the National Eczema Society and the Royal College of Nursing to enhance confidence the content was real and trustworthy as required by lay co-creators. The ripple effect of interventions is illustrated in Image 1.

KMb materials	Recipients
Postcards and posters with key messages and supplementary information, for example 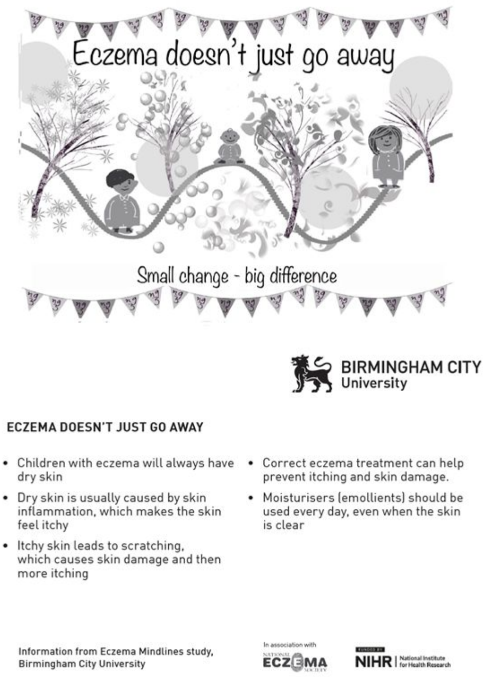	HCPs in local area including GPs, GP trainees, practice nurses, health visitors, community pharmacists and pharmacy counter assistants<br><br>Displayed in local infant and primary schools, libraries, places of worship, GP practices, community pharmacies
Mindline informed educational sessions, led by DNS	Health visitors, community public health nurses (n=36), GPs (n=18), practice nurses (n=8)

For peer review only - http://bmjopen.bmj.com/site/about/guidelines.xhtml

Shopping centre pod for rapid consultations with two DNSs	Consultations with customers (n=94)
In person and online story reading and activity sessions in<br>• nurseries and primary schools<br>• places of worship	Children (n=86), teachers and teaching assistants (n=11)<br>Children and parents (n=~50)
Eczema mindlines website	Freely available online
The Dragon in My Skin book the-dragon-in-my-skin-132634726304040297.pdf (windows.net)	Freely available online<br>Hard copies distributed to primary schools (n=792) with links to all other Dragon resources
Dragon workshops	Children with eczema (n=10), their parents, an author and professional orchestra members
Dragon premiere	Children with eczema, their parents, professional orchestra members and invited guests with an interest in eczema including practitioners and members of eczema organisations (n=62)
Dragon book the-dragon-in-my-skin-132634726304040297.pdf (windows.net)	Freely available online<br>Hard copies distributed to primary schools (n=792)
Dragon Teacher resource pack tdims-workpack-v2-long-132693393982395364.pdf (windows.net)	Freely available online
Dragon Animation The Dragon In My Skin - School of Health Sciences | Birmingham City University (bcu.ac.uk)	Freely available online
Dragon translations	Will be freely available online
Eczema mindlines documentary https://youtu.be/C4d_yxvHVPk	Freely available online

Table 4: KMb interventions

Stage 3: Analysis of intervention impact

Aim

To systematically evaluate the impact of co-creating and delivering KMb interventions to alter and enhance mindlines and improve childhood eczema care.

Design

Social Impact Framework evaluation

For peer review only - http://bmjopen.bmj.com/site/about/guidelines.xhtml

Data collection

Data collection was multi-factorial, data sources and collection methods are summarised in Table 5.

Data source	Data collection method
HCPs including GPs, GP trainees, practice nurses, health visitors, community pharmacists and pharmacy counter assistants	Qualitative interviews, informal conversations, email feedback
Health visitor and community practitioners	Post session evaluation and subsequent email feedback
Children, parents and artists engaged in Dragon work	Observation, informal conversations, email feedback
Attendees at world premiere of Dragon event	Immediate comments, Zoom chat, follow-up emails
Teachers and student teachers	Online survey pre and post using Dragon resources in practice, informal conversations
Charitable organisations	Email, informal conversations
Professional organisations	Email feedback, testimonial
Social media	Metrics and testimonials
Researcher	Reflective diary of observation and experiences at all stages

Table 5: Summary of data sources and data collection methods

Data analysis

Data analysis was guided by the Social Impact Framework (SIF) (12, 24) to capture multi-level processes, impacts and key mechanisms of our KMb activities. We collated data from all sources including transcripts of audio recorded interviews, feedback from online meetings and events, testimonials, email correspondence, artefacts (such as children's drawings), researcher observation and conversations, online surveys and metrics. These data could not and should not be separated from knowledge of the study design and the condition and creation of data (38). The language and 'things' were inseparable (39), and so were analysed together. Two authors (FC and RP) iteratively read, thought and wrote about and discussed the data in its totality to identify impact (40).

Reflexivity

A reflexive stance was maintained for the duration of the study acknowledging both the complexities of the world and researcher entanglement with the fullness of the research process (41) and our preconceived understandings.

Patient and Public Involvement

Lay people were involved in the development of the research question, planning and delivering the study and co-creation and evaluation of KMb interventions.

For peer review only - http://bmjopen.bmj.com/site/about/guidelines.xhtml

Results

Results are documented according to the four outcomes.

Outcome 1: What is the impact of this study on individuals and groups?

Impact on individuals and groups was significant. Members of the co-creation group (n=22 lay people, practitioners and researcher) reported new understandings of eczema care from the 'other' perspective, *"conversations show how little lay people and HCPs understand each other's worlds and how interested they are in getting new insights" [researcher].* Participants demonstrated a new respect for the skills, knowledge and experiences of others and similarly gained a deeper understanding of the challenges and constraints of others. Co-creation enabled cross-fertilisation of ideas alongside a realisation of the power each person has to make a difference. Lay members found new ways of, and confidence in, communicating with practitioners and researchers.

At the time of writing engagement with the mindlines webpage, Facebook and Twitter is modest (Table 6) and suggests that personal approaches may be more impactful.

- All time views on the mindlines webpage to date 1146, split between documentary (n=386), animation (n=696) and knowledge nuggets (n=54).
- Direct views to the video guides n=121. View quality was high, people spent on average of five minutes across all pages.
- Uptake on Facebook and Twitter achieved nine and 121 followers respectively.
- Animation views for the five messages were: i) eczema doesn't just go away (n=270), ii) eczema is more than just dry skin (n=684), iii) moisturisers are for every day (n=58), iv) steroid creams are okay when you need them (n=223) and v) you know your child's eczema best (n=not recorded).

Table 6: Engagement with mindlines website

Strategically placed posters and postcards impacted on the thinking of staff. Many either had or knew others who had eczema and thought the resources would be helpful. Pharmacy counter assistants supported adding a pack of postcards with all dispensed eczema topical treatments. A

For peer review only - http://bmjopen.bmj.com/site/about/guidelines.xhtml

shopping centre pod set up for rapid consultations with two Dermatology Nurse Specialists (DNSs) attracted 94 people in one day. Immediacy, advice from an expert and personalisation of the five key messages was highly valued in verbal feedback. It provided a basis for individuals to change their eczema self-management (although it is not possible to know whether this change was enacted). Children engaged enthusiastically with story reading and activity sessions Teachers reported ongoing conversations about how it must feel to live with eczema and increased empathy both themselves and amongst children. Attendance at mindline informed eczema sessions for HCPs was higher than anticipated. Evaluation was overwhelmingly positive, particularly in terms of contextual relevance and applicability to own practice, for example "it *stuck in my mind, direct relation to patients, the "hook" to apply to own practice* [GP].

The Dragon book was described as "*beautifully profound in its simplicity*" [orchestra member] and "*superb, I love the story and the pictures, what a lovely way for children with eczema to be able to see how they can tame their dragon and to have its impact validated in such a wonderful way!"* [lay person]. Through word-of-mouth connections around 200 further books were sent to other educators and HCPs. A Dermatology Specialist Nurse shared the book with children attending her clinic and wrote "*They love it, the children feel they have more control and it have made them feel special ………. this for me has been one of the best tools to use*".

Children enjoyed the Dragon online co-creation sessions "[child's name] *always looked forward to her sessions on zoom and you all made it so easy to engage and be confident. It's almost a shame the sessions are finished!*". Parents commented "*You guys do a great job at engaging the kids …… because you're after their input they're invested early on*" and also valued validation of the realities of living with eczema. They were proud to be part of the online premiere. Feedback from this included

- *"emotional…… this really helped me see what [my child] is feeling" [parent].*
- One mother described her daughter's anger at having to manage the condition was struck *"to hear that [anger] validated in a book for [my child] to understand"*
- *"It's beautiful. And as someone with a dragon since day one in life and struggling at the moment with it, I was especially moved by this." [teacher]*
- *"So often it's seen as 'just eczema'… it's nice to have something that shows how hard it is"* [parent],
- *"loved the way you haven't shied away from the difficult and painful experiences and feelings children have about eczema"* [charitable organisation]

For peer review only - http://bmjopen.bmj.com/site/about/guidelines.xhtml

- *"you've taken a debilitating but common and overlooked problem and made it come alive … I found it very moving"* [HCP].

Parents noted the benefits of greater awareness of eczema amongst teachers and other children through widespread sharing of the Dragon resources *"I am so glad that this will now be shared in schools to raise awareness amongst children of what some of their friends are going through"* [parent], *"Just awesome. That's so good. I'll send it to [child's name] teacher because there's a little girl in his class really suffering" [parent].* The YouTube animation has been viewed 1,378 times with 61.2% of views outside the United Kingdom, the average view duration was 3 minutes and 29 seconds and 22% of viewers watched all content relating to the five key messages (up to 9mins 45secs).

Outcome 2: What changes in behaviour, practice and research have occurred due to their involvement?

Children and parents

Tangible changes in behaviour and practice were described. Some children and parents recounted more concordance with treatment, for example *"*[child's name] *wanted me to tell you that she has put her spray on all over to look after her dragon"* [parent]. However, for most the more important change was the recognition that others gave to their child's eczema "*so often it's seen as 'just eczema' … it's nice to have something that shows how hard it is*" [parent].

Health care practitioners

Practitioners reported not necessarily learning anything new but rather 'fine-tuning' their mindlines and changing in thinking for example Dragon "*validates experiences and feelings …… shows we understand*" [HCP]. Examples of simple but effective practice changes included:

- *"the three main things that I took away from it were using one application of steroids is just as good as two. Go big early with the steroid and go greasier as well, really, rather than have a kind of hierarchy, going for a greasier emollient earlier rather than wait"* [nurse] several months post-intervention the same nurse reported "*it's certainly made me more confident in prescribing, really. I don't think patients are coming back as much, I think actually going bigger earlier has a positive effect, really"*
- *"I think probably we've often a bit mean with it …. I've double checked that they've got enough of the emollients"* [GP].

For peer review only - http://bmjopen.bmj.com/site/about/guidelines.xhtml

- *"I readily use the information on my contacts at home visits and during clinic times, it is valuable to my practice and aids my prescribing for children with skin conditions, and when to refer"* [HV].
- *"I use [postcards] in practice and in teaching my students about care of the skin on a regular basis"* [HV].

Teachers

Use of resources led to more understanding from teachers and peers, "*used it [book] with her individually to help the child manage her emotions and consider how she could manage her condition in school and that now the little girl picks the book up to read whenever she needs a 'comfort blanket' moment*" [teacher]. Teachers using Dragon resources conveyed value through words and images. "*The children had some really mature discussion during this lesson and I have to say I was impressed, a couple of children with eczema were heavily involved in this and told other pupils some of their experiences (without being prompted or pressured to do so)" [teacher]. "I have looked at this story with children in my class and they absolutely loved it. I created a hook where I had an animated dragon that came into our classroom and left some footprints and burnt paper and it got the children wondering why we had a dragon come in. The children then created their own story maps and understood the concept of eczema, as we have two girls who suffer from it. It was amazing*", see Image 1. The implication from teacher feedback was that other children developed greater empathy for peers with eczema with the suggestion that this approach would reduce unkindness and bullying.

Outcome 3: What mechanisms have enabled these impacts or changes to occur?

Multiple mechanisms enabled impact including: simplicity and consistency of messages adapted to audience, flexibility, opportunism and perseverance, personal interconnectivity and acknowledgment of emotion.

Simplicity and consistency of messages adapted to audience

All KMb was underpinned by the five simple, key messages. Although not new these messages are at the heart of most eczema care with the mantra being "get control-keep control" through use of topical corticosteroids when needed and regular and consistent application of emollients. Consistent, cross boundary messaging was intended to bring about shared language and understanding on which to base more equal eczema consultations. The role of the DNSs was pivotal.

For peer review only - http://bmjopen.bmj.com/site/about/guidelines.xhtml

Mindline informed teaching engaged HCPs, using their own stories to *"hang things on"* allowing immediate contextualisation and application of new knowledge. Equally having an expert with a wealth of current clinical and research knowledge and a repertoire of anecdotes made session rich, relevant and real. Lay people relished the opportunity to get on-the-spot expert, personal advice at the shopping centre. Immediacy was key to success. The five key messages provided a scaffold for each consultation, essentially each person received the same key information, but the DNSs, trusted sources of information, skilfully adapted and integrated messages to make them meaningful and useful to each individual.

The Dragon offered the five messages in child-friendly formats which addressed eczema care from a positive, proactive standpoint rather than the more usual problematisation of the condition. Dragon related KMb activity has grown exponentially mainly by word of mouth supplemented by sharing in newsletters, magazines and websites. Numerous requests for resources have been received from HCPs and teachers. For example, one National organisation with a mission to transform localities with creativity and culture wrote the Dragon is "*Beautifully composed, created, animated and such a positive piece for children and young people to be involved in when eczema can be so hard"* and went on share the resources across wide-ranging networks. An attendee at the Dragon premiere descried the resources as "*incredible"* and shared them with every primary school and primary care practice in one region. An education leader who heard about the Dragon through a personal contact wrote "*I am delighted to be able to share these resources with our 87 mental health leads …. as I believe that this resource can support reducing the stigma linked to eczema, often born out of ignorance of the condition".* Through HCP contacts Dragon resources are in the process of being translated and culturally adapted into French and Portuguese.

Flexibility, opportunism and perseverance

Diligent, persistent, adaptable and proactive knowledge brokering was an essential element of enabling impact, as was perseverance in the face of practical and process constraints. Perseverance and patience were required in managing bureaucracy in setting up events and when events were cancelled at the last minute and needed to be rebooked. Some people rejected my offer outright including one children's play venue manager who would not support anything that suggested steroid creams were okay when you need them and a leisure centre manager who stated the messages were *"not suitable"*. Effective knowledge brokering also relied on i) building robust and enduring relationships with leading eczema charities and professional organisations and securing their

For peer review only - http://bmjopen.bmj.com/site/about/guidelines.xhtml

endorsement ii) engaging with influencers, authority figures and decision-makers and iii) openness to collaborative working across new networks.

Personal interconnectivity

Personal interconnectivity was a key factor in sharing messages (Figure 2). Through personal contacts, email and telephone calls multiple individuals and organisations were contacted offering to share the key eczema messages using approaches tailored to each venue. Personal networks were effective door openers, for example a practice manager introduced me to an Imam, who introduced me to a pharmacist, and so it went on, allowing me to access many groups I would not otherwise have reached. Equally it was the starting point and central in developing, sharing and using Dragon resources. The idea was influenced by conversations with a patient group who highlighted the need to make teachers and children more aware of eczema and mainstream it rather than using existing problematising approaches such as having a special assembly on the condition. A chance conversation with a colleague led to development of the Dragon teacher resource pack. Consideration was given to the limitations of personal connections. We all inherently move in our own limited circles however, we strived for inclusivity through situating our work 'out there' and using the ripple effect to meet new and unexpected allies.

Insert Figure 2: Illustration of interconnectivity

Acknowledgment of emotion

Tapping into emotions amplified the impact of KMb activity on altering and enhancing mindlines. For HCPs relating knowledge to individual patients and their families was more powerful than generic teaching and sessions also gave space to express the frustrations of eczema care and collaboratively seek more positive approaches. For lay consultations being "*listened to*" as a whole person was key. Numerous Dragon comments focused on emotion as much as content, for example a teacher wrote "*It is such a wonderful concept that will make such a difference to children with and without eczema. I know me and my daughter would have felt much happier at school if we'd had something like this".* An experienced HCP commented "*You've taken a debilitating but common and overlooked problem and made it come alive! I loved it all and found it very moving"* and many parents echo the sentiment of a charity leader "*Loved the way you haven't shied away from the difficult and painful experiences and feelings children have about eczema*".

For peer review only - http://bmjopen.bmj.com/site/about/guidelines.xhtml

Outcome 4: What are the recommendations and questions arising from this research?

This research has important implications in terms of future KMb activity.

- Firstly, altering and enhancing mindlines across patient-practitioner-wider society boundaries is possible and effective in changing behaviour / practice. Mindlines inherently made sense to all participants. Existing evidence was used to inform development of key, simple messages that were shared using creative and contextually adroit (19) formats that were relevant and applicable for end users.
- Secondly, knowledge brokering may start with one person but building up networks of knowledge brokers is essential. In this instance the process was organic and was strengthened by openness to unexpected opportunities. In future thought must be given to potential networks but equally researchers need to be open to and actively seeking new possibilities.
- Finally, the Social Impact Framework offers a robust and iterative approach to planning, mapping and evidencing impact. 'Proving' the value of KMb is not and never will be straightforward. However, adoption of the SIF offers a step-change in demonstrating wide-ranging impact of KMb activity.

Discussion

The aim of this study was to evaluate the impact of using KMb interventions to alter and enhance mindlines and improve childhood eczema care. Five key co-created messages were shared locally, nationally and internationally using a range of tailored and creative resources. Crucially the messages were transmitted across lay-practitioner-wider society boundaries to enhance development of shared understanding and language. KMb interventions were developed to meet lay peoples need for 'trust' and 'realness' and HCPs requirement for practical, locally relevant, hints and tips, tailored, 'no faff' approaches. Multiple mechanisms enabled impact including: simplicity and consistency of messages adapted to audience, flexibility, opportunism and perseverance, personal interconnectivity and acknowledgment of emotion. The Dragon offered the five messages in child-friendly formats which addressed eczema care from a positive, proactive standpoint rather than the more usual problematisation of the condition. The evidence presented demonstrates the resonance that the work as a whole had with people living eczema and those providing care. Recognition of the challenges and use of contextually relevant interventions for both appear to have increased receptivity and integration of new knowledge into everyday care. This study illustrates the complexities of assessing impact of KMb activity and offers an approach that captures impact at multiple levels and from different perspectives.

For peer review only - http://bmjopen.bmj.com/site/about/guidelines.xhtml

This original study is one of the first to methodically evaluate the impact of using KMb interventions to alter and enhance mindlines across patient-practitioner-wider society boundaries. The Social Impact Framework has a strong theoretical base and offers structure and depth for evaluating impact. Use of the SIF has enabled reflection on the complex web of impact from a range of perspectives which may be overlooked if using more traditional measures. Reporting is in accordance with the consolidated criteria for reporting qualitative research (42).

Assessment of KMb impact revealed sharing at local level allowed deep engagement across the community, but only from those who chose to engage. Local KMb activity was labour intensive and engagement of influential individuals in different organisations was essential, scale up possibilities are therefore limited. Dragon resources were influential in achieving impact. Although currently aimed at an English-speaking audience, the Dragon is now being translated and culturally adapted leading to significant opportunities for international sharing and benefit. We acknowledge that it is unlikely that all impact has been captured and indeed, new ripples of impact are ongoing. The work has, to an extent, taken on a life of its own and been spread through personal contacts and existing networks. We are mindful that this work will have made a contribution to changes in practice or behaviour but cannot definitively claim change, but again the evidence presented suggests changes in thinking that are likely to influence actions.

Methodical assessment of the impact of KMb, activity is scarce (43) despite allied literature pointing to the need to build understanding (44) and competence (45) in this arena. Impact is a contested term, sometimes conceptualised as a linear process (46) in which impact is *directly attributable* to generation and dissemination of new knowledge (47). In the present study impact was viewed from the wide-ranging lens of the SIF. We are mindful that there are many other influences on eczema care and that this work offers a *contribution* to change (48). Application of the SIF has allowed a nuanced understanding of the depth and breadth of impact of KMb activities and contributed to the much-needed development of KMb theory (44). The SIF although primarily directed to evaluating co-production, offered a structured approach to reflect on micro-macro levels, processes, impacts and mechanisms of the KMb activity and map the winding pathway of incremental and often subtle changes which are readily overlooked.

The KMb interventions used to share simple consistent messages, co-created by end users is congruent with current thinking about challenges of KMb. Extant literature points to i) information

For peer review only - http://bmjopen.bmj.com/site/about/guidelines.xhtml

overload for HCPs (49) and lay people (50), ii) inconsistent advice regarding eczema care (51), iii) poor quality information and limited confidence in assessing veracity of available information for lay people (52), iv) the need to consistently work with end users to increase uptake of knowledge (53) and v) the value of promoting shared language and understandings and thus support shared decision making and self-management (54). Gabbay and Le May (19) identify the inter-relationship of patient-practitioner mindlines and hence the need to change mindlines in parallel. However, few studies have considered KMb across lay-practitioner-wider society boundaries (21).

Knowledge brokers as intermediaries between researchers and practitioners are well established in healthcare as evidenced in recent reviews (55, 56). Nevertheless, the role can be problematic with some brokers challenged by role ambiguity and the need for a multidimensional skill set (57). In the present study the broker being a researcher and nurse and having lived experience of eczema minimised these tensions and were of distinct benefit in the relationship brokering component of the role (58). Over time others took up brokering activity, which enhanced capacity to move evidence to practice (57).

Systematic analysis of KMb activity has highlighted multiple mechanisms influencing impact which may be applied in future KMb work. In the present study key processes included: i) engagement of key stakeholders and end users, ii) appreciative engagement, creating opportunities for engagement, valuing unique individual contributions and respectful working, iii) diligent, persistent and proactive knowledge brokering, iv) sustained supportive relationships, v) use of iterative flexible processes, adjustment to contextual challenges and changing circumstances and vi) creativity and use of diverse media. The KMb materials provide lay people HCPs and teachers with evidence-based resources to use and share with others. We also offer a novel approach to systematically evaluating KMb activity which builds much needed theory alongside practical application. There is still much work to be done to better understand the impact of knowledge mobilisation strategies specifically those striving to bridge lay-practitioner-wider society boundaries to improve care.

review only

Conclusion

This study is one of the first to systematically assess the impact of KMb interventions designed to alter and enhance mindlines across lay-practitioner-wider society boundaries. The Social Impact Framework has been used to transparently map the complex web of impact from a range of perspectives which may be overlooked if using more traditional measures. Crucially impact has included tangible changes in childhood eczema care practice and self-management and 'mainstreamed' the condition to enhance understanding of children and teachers. It brings to the

For peer review only - http://bmjopen.bmj.com/site/about/guidelines.xhtml

fore new understandings of key mechanisms underpinning effective KMb practice. The challenge now is to test this approach to assess the impact of other types of KMb interventions.

For peer review only

For peer review only - http://bmjopen.bmj.com/site/about/guidelines.xhtml

Contributionship statement

FC: Led the research, including study design, data acquisition and interpretation and writing this article. She is accountable for all aspects of the work in ensuring that questions related to the accuracy or integrity of any part of the work are appropriately investigated and resolved.

SL: Contributed to study design, critical revision and gave approval for submission

RP: Contributed to study design, data acquisition and interpretation, critical revision and gave approval for submission

JVO: Contributed to data acquisition, critical revision and gave approval for submission

Competing interests

The authors have no competing interests to declare

Funding

This report is independent research arising from a Knowledge Mobilisation Research Fellowship, Professor Fiona Cowdell, KMRF-2015-04-004 supported by the National Institute for Health Research. The views expressed in this publication are those of the authors and not necessarily those of the NHS, the National Institute for Health Research, Health Education England or the Department of Health.

Data sharing statement

Data are available upon reasonable request to the corresponding author

Statement of Ethics Approval

This manuscript presents evaluation data only and so does not require ethics approval.

Acknowledgments

We extend our thanks to all who have taken part in this study and to Kate Beckett who has kindly shared her knowledge and expertise when commenting on drafts of this manuscript. Thanks also to Jay Nolan Latchford and Michaela Tait for creating images.

For peer review only

References

1. Abuabara K, Ye M, McCulloch CE, Sullivan A, Margolis DJ, Strachan DP, Paternoster L, Yew YW, Williams HC, Langan SM. Clinical onset of atopic eczema: results from 2 nationally representative British birth cohorts followed through midlife. Journal of Allergy and Clinical Immunology. 2019 Sep 1;144(3):710-9.
2. Teasdale E, Muller I, Sivyer K, Ghio D, Greenwell K, Wilczynska S, Roberts A, Ridd MJ, Francis N, Yardley L, Thomas KS. Views and experiences of managing eczema: systematic review and thematic synthesis of qualitative studies. British Journal of Dermatology. 2021 Apr;184(4):627-37.
3. Le Roux E, Powell K, Banks JP, Ridd MJ. GPs' experiences of diagnosing and managing childhood eczema: a qualitative study in primary care. British Journal of General Practice. 2018 Feb 1;68(667):e73-80.
4. National Institute for Heath and Care Excellence. Atopic eczema in under 12s: diagnosis and management. Clinical guideline (CG57) updated March 2021 Overview | Atopic eczema in under 12s: diagnosis and management | Guidance | NICE Accessed 24.01.22
5. National Institute for Heath and Care Excellence, Clinical Knowledge Summary, Eczema-atopic Eczema - atopic | Health topics A to Z | CKS | NICE Accessed 24.01.22
6. Ellis J, Boger E, Latter S, Kennedy A, Jones F, Foster C, Demain S. Conceptualisation of the 'good'self-manager: A qualitative investigation of stakeholder views on the self-management of long-term health conditions. Social Science & Medicine. 2017 Mar 1;176:25-33.
7. Williams O, Sarre S, Papoulias SC, Knowles S, Robert G, Beresford P, Rose D, Carr S, Kaur M, Palmer VJ. Lost in the shadows: reflections on the dark side of co-production. Health Research Policy and Systems. 2020 Dec;18:1-0.
8. Greenhalgh T, Papoutsi C. (2018) Studying complexity in health services research: desperately seeking an overdue paradigm shift. *BMC Med.* 16 (95) doi:10.1186/s12916-018-1089-4
9. INVOLVE Guidance on co-producing a research project 2018 Copro_Guidance_Feb19.pdf (invo.org.uk) Accessed 24.01.22
10. Cowdell F, Dyson J, Sykes M, Dam R, Pendleton R. How and how well have older people been engaged in healthcare intervention design, development or delivery using co-methodologies: A scoping review with narrative summary. Health & Social Care in the Community. 2020 Oct 25.
11. Greenhalgh T, Jackson C, Shaw S, Janamian T. Achieving research impact through co-creation in community-based health services: literature review and case study. The Milbank Quarterly. 2016 Jun;94(2):392-429.
12. Beckett K, Deave T, McBride T, le May Andrée A, Gabbay J, Kapoulas U, Long A, Warburton G, Wogan C, Cox L, Thompson J. Using Forum Theatre to mobilise knowledge and improve NHS care: The Enhancing Post-injury psychological Intervention and Care (EPPIC) study. Evidence and Policy. https://doi.org/10.1332/174426421X16420902769508
13. Haynes A, Rowbotham S, Grunseit A, Bohn-Goldbaum E, Slaytor E, Wilson A, Lee K, Davidson S, Wutzke S. Knowledge mobilisation in practice: An evaluation of the Australian Prevention Partnership Centre. Health research policy and systems. 2020 Dec;18(1):1-7.
14. Kislov R, Waterman H, Harvey G, Boaden R. Rethinking capacity building for knowledge mobilisation: developing multilevel capabilities in healthcare organisations. Implementation Science. 2014 Dec;9(1):1-2.
15. Powell A, Davies H, Nutley S. Missing in action? The role of the knowledge mobilisation literature in developing knowledge mobilisation practices. Evidence & Policy: A Journal of Research, Debate and Practice. 2017 13(2):201-23.

For peer review only - http://bmjopen.bmj.com/site/about/guidelines.xhtml

16. Marshall M, Eyre L, Lalani M, Khan S, Mann S, de Silva D, Shapiro J. Increasing the impact of health services research on service improvement: the researcher-in-residence model. Journal of the Royal Society of Medicine. 2016 Jun;109(6):220-5.
17. Ward V. Why, whose, what and how? A framework for knowledge mobilisers. Evidence & Policy. 2017 13(3):477-497 . https://doi.org/10.1332/174426416X14634763278725
18. Davies HTO, Powell AE, Nutley SM. Mobilising knowledge to improve UK health care: learning from other countries and other sectors – a multimethod mapping study. NIHR Journals Library, Southampton (UK); 2015. PMID: 26110190.
19. Gabbay J, Le May A. Practice-based evidence for healthcare: clinical mindlines. Abingdon, Routledge; 2011.
20. Nonaka I, Toyama R, Konno N. SECI, Ba and leadership: a unified model of dynamic knowledge creation. Long range planning. 2000 Feb 1;33(1):5-34.
21. Appleby B, Cowdell F, Booth A. Knowledge mobilization in bridging patient-practitioner-researcher boundaries: A systematic integrative review. Journal of Advanced Nursing. 2021 Feb;77(2):523-36.
22. Weiss D, Lillefjell M, Magnus E. Facilitators for the development and implementation of health promoting policy and programs–a scoping review at the local community level. BMC Public Health. 2016 16(1):1-5.
23. Worton S, Loomis C, Pancer S, Nelson G, Peters RD. Evidence to impact: A community knowledge mobilisation evaluation framework. Gateways: International Journal of Community Research and Engagement. 2017 10:121-42.
24. Beckett K, Farr M, Kothari A, Wye L, Le May A. Embracing complexity and uncertainty to create impact: exploring the processes and transformative potential of co-produced research through development of a social impact model . Health research policy and systems. 2018 16(1):1-8.
25. Cowdell F. Knowledge mobilisation: an ethnographic study of the influence of lay mindlines on eczema self-management in primary care in the UK. BMJ open. 2018 Aug 1;8(8):e021498.
26. Cowdell F. Knowledge mobilisation: an ethnographic study of the influence of practitioner mindlines on atopic eczema self-management in primary care in the UK. BMJ open. 2019 Jul 1;9(7):e025220.
27. Cowdell F. Knowledge mobilisation: an exploratory qualitative interview study to confirm and envision modification of lay and practitioner eczema mindlines to improve consultation experiences and self-management in primary care in the UK. BMJ open. 2019 Jun 1;9(6):e028225.
28. Cowdell F, Ahmed T, Layfield C. Knowledge mobilisation: a UK co-creation study to devise strategies to amend lay and practitioner atopic eczema mindlines to improve consultation experiences and self-management practices in primary care. BMJ open. 2020 Sep 1;10(9):e036520.
29. Carlan NA, Kramer DM, Bigelow P, Wells R, Garritano E, Vi P. Digging into construction: Social networks and their potential impact on knowledge transfer. Work. 2012 Jan 1;42(2):223-32.
30. Thompson MR, Schwartz Barcott D. The role of the nurse scientist as a knowledge broker. Journal of Nursing Scholarship. 2019 Jan;51(1):26-39.
31. Nishida K, Dilworth DA. Fundamental Problems of Philosophy the World of Action and the Dialectical World. Translated with an Introd. By David A. Dilworth. 1970.
32. Nishida K. An Inquiry into the Good, as translated by Abe, M. and C. Ives.(1990). New Haven./London: Yale University, 1921.
33. Shimizu H. Ba-principle: new logic for the real-time emergence of information. Holonics 1995;5:67–79.

For peer review only - http://bmjopen.bmj.com/site/about/guidelines.xhtml

34. Nambozi, G (2014) An evaluation of community based university nursing education programme and stakeholders' experiences. https://hydra.hull.ac.uk/assets/hull:14394a/content (accessed 18 May 2021).
35. Newman ME. Ego-centered networks and the ripple effect. Social Networks. 2003 Jan 1;25(1):83-95.
36. Chin JH, Mansori S. Social marketing and public health: A literature review. Journal of Marketing Management and Consumer Behavior. 2018 May 20;2(2).
37. Evans WD, Christoffel KK, Necheles JW, Becker AB. Social marketing as a childhood obesity prevention strategy. Obesity. 2010 Feb 1;18(n1s):S23.
38. Marn TM, Wolgemuth JR. Applied Qualitative Data Analysis After the Ontological Turn. Qualitative Report. 2021 Jun 1;26(6).
39. Lather P, St. Pierre EA. Post-qualitative research. International journal of qualitative studies in education. 2013 Jul 1;26(6):629-33.
40. Mazzei LA, Jackson AY. Complicating voice in a refusal to "let participants speak for themselves". Qualitative inquiry. 2012 Nov;18(9):745-51.
41. Østern TP, Jusslin S, Nødtvedt Knudsen K, Maapalo P, Bjørkøy I. A performative paradigm for post-qualitative inquiry. Qualitative Research. 2021 Jul 7:14687941211027444.
42. Tong A, Sainsbury P, Craig J. Consolidated criteria for reporting qualitative research (COREQ): a 32-item checklist for interviews and focus groups. *Int J Qual Health Care* 2007;19(6):349-357.
43. Labbé D, Mahmood A, Miller WC, Mortenson WB. Examining the Impact of Knowledge Mobilization Strategies to Inform Urban Stakeholders on Accessibility: A Mixed-Methods study. International journal of environmental research and public health. 2020 Jan;17(5):1561.
44. Powell A, Davies H, Nutley S. Missing in action? The role of the knowledge mobilisation literature in developing knowledge mobilisation practices. Evidence & Policy: A Journal of Research, Debate and Practice. 2017 May 19;13(2):201-23.
45. Bayley JE, Phipps D, Batac M, Stevens E. Development of a framework for knowledge mobilisation and impact competencies. Evidence & Policy: A Journal of Research, Debate and Practice. 2018 Nov 26;14(4):725-38.
46. Abma TA, Cook T, Rämgård M, Kleba E, Harris J, Wallerstein N. Social impact of participatory health research: collaborative non-linear processes of knowledge mobilization. Educational action research. 2017 Aug 8;25(4):489-505.
47. Morton S, Wilson S, Inglis S, Ritchie K, Wales A. Developing a framework to evaluate knowledge into action interventions. BMC health services research. 2018 Dec;18(1):1-3.
48. Morton S. Progressing research impact assessment: A 'contributions' approach. Research Evaluation. 2015 Oct 1;24(4):405-19.
49. Nagtegaal R, Tummers L, Noordegraaf M, Bekkers V. Nudging healthcare professionals towards evidence-based medicine: a systematic scoping review. Journal of Behavioral Public Administration. 2019 Oct 2;2(2).
50. Khaleel I, Wimmer BC, Peterson GM, Zaidi ST, Roehrer E, Cummings E, Lee K. Health information overload among health consumers: a scoping review. Patient education and counseling. 2020 Jan 1;103(1):15-32
51. Teasdale E, Muller I, Sivyer K, Ghio D, Greenwell K, Wilczynska S, Roberts A, Ridd MJ, Francis N, Yardley L, Thomas KS. Views and experiences of managing eczema: systematic review and thematic synthesis of qualitative studies. British Journal of Dermatology. 2021 Apr;184(4):627-37.
52. Mueller SM, Hongler VN, Jungo P, Cajacob L, Schwegler S, Steveling EH, Thomas ZR, Fuchs O, Navarini A, Scherer K, Brandt O. Fiction, falsehoods, and few facts: cross-sectional study on

the content-related quality of atopic eczema-related videos on YouTube. Journal of medical Internet research. 2020 Apr 24;22(4):e15599.
53. Slattery P, Saeri AK, Bragge P. Research co-design in health: a rapid overview of reviews. Health research policy and systems. 2020 Dec;18(1):1-3.
54. Gruffydd-Jones K, Hansen K. Working for better asthma control: how can we improve the dialogue between patients and healthcare professionals?. Advances in Therapy. 2020 Jan;37(1):1-9.
55. Scarlett J, Forsberg BC, Biermann O, Kuchenmüller T, El-Khatib Z. Indicators to evaluate organisational knowledge brokers: a scoping review. Health research policy and systems. 2020 Dec;18(1):1-3.
56. Ayatollahi H, Zeraatkar K. Factors influencing the success of knowledge management process in health care organisations: a literature review. Health Information & Libraries Journal. 2020 Jun;37(2):98-117.
57. Kislov R, Wilson P, Boaden R. The 'dark side'of knowledge brokering. Journal of health services research & policy. 2017 Apr;22(2):107-12.
58. Wye L, Cramer H, Carey J, Anthwal R, Rooney J, Robinson R, Beckett K, Farr M, le May A, Baxter H. Knowledge brokers or relationship brokers? The role of an embedded knowledge mobilisation team. Evidence & Policy: A Journal of Research, Debate and Practice. 2019 May 1;15(2):277-92.

For peer review only - http://bmjopen.bmj.com/site/about/guidelines.xhtml

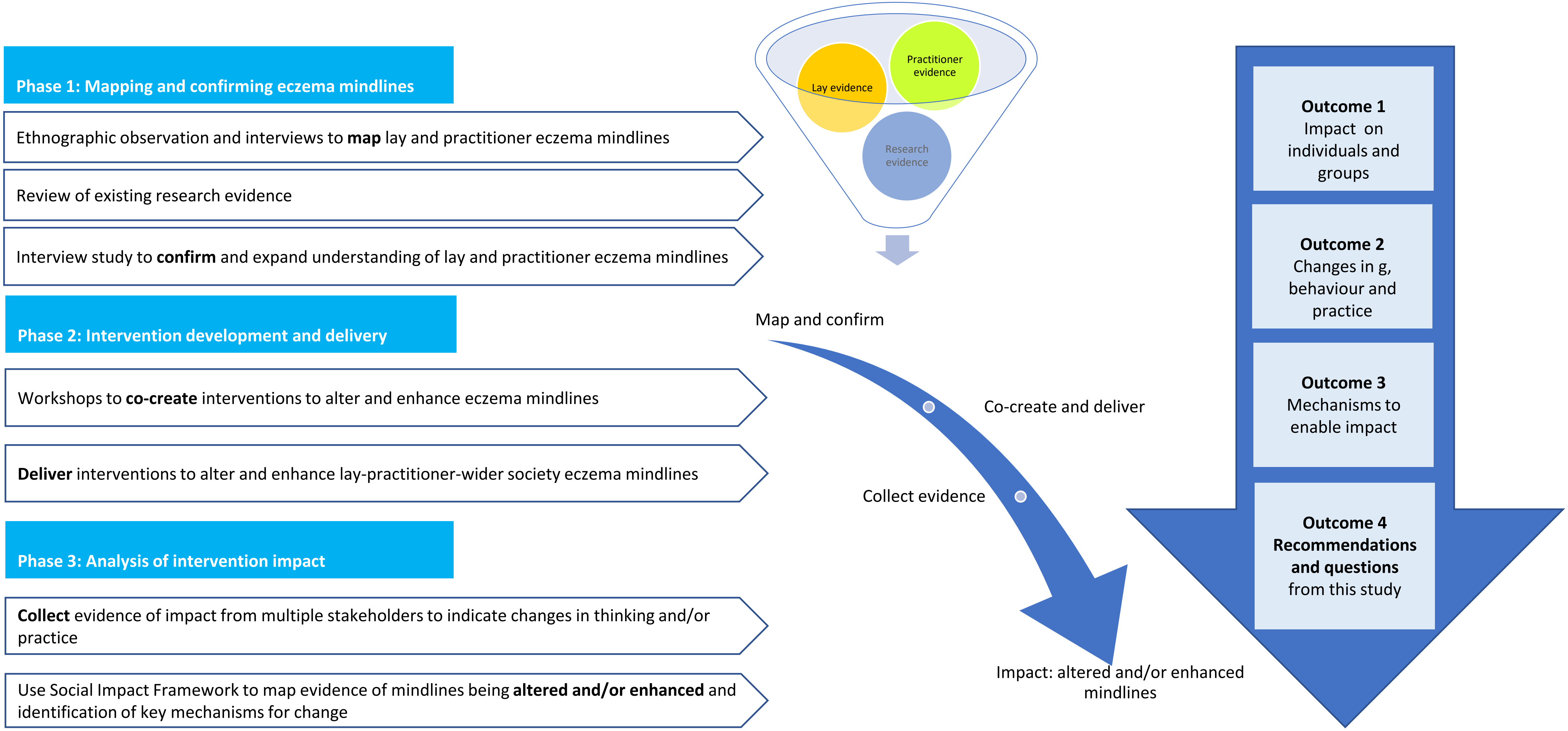
Phase 1: Mapping and confirming eczema mindlines
Ethnographic observation and interviews to map lay and practitioner eczema mindlines
Review of existing research evidence
Interview study to confirm and expand understanding of lay and practitioner eczema mindlines
Phase 2: Intervention development and delivery
Workshops to co-create interventions to alter and enhance eczema mindlines
Deliver interventions to alter and enhance lay-practitioner-wider society eczema mindlines
Phase 3: Analysis of intervention impact
Collect evidence of impact from multiple stakeholders to indicate changes in thinking and/or practice
Use Social Impact Framework to map evidence of mindlines being altered and/or enhanced and identification of key mechanisms for change
Practitioner evidence
Lay evidence
Research evidence
Map and confirm
Co-create and deliver
Collect evidence
Impact: altered and/or enhanced mindlines
Outcome 1
Impact on individuals and groups
Outcome 2
Changes in g, behaviour and practice
Outcome 3
Mechanisms to enable impact
Outcome 4
Recommendations and questions
from this study

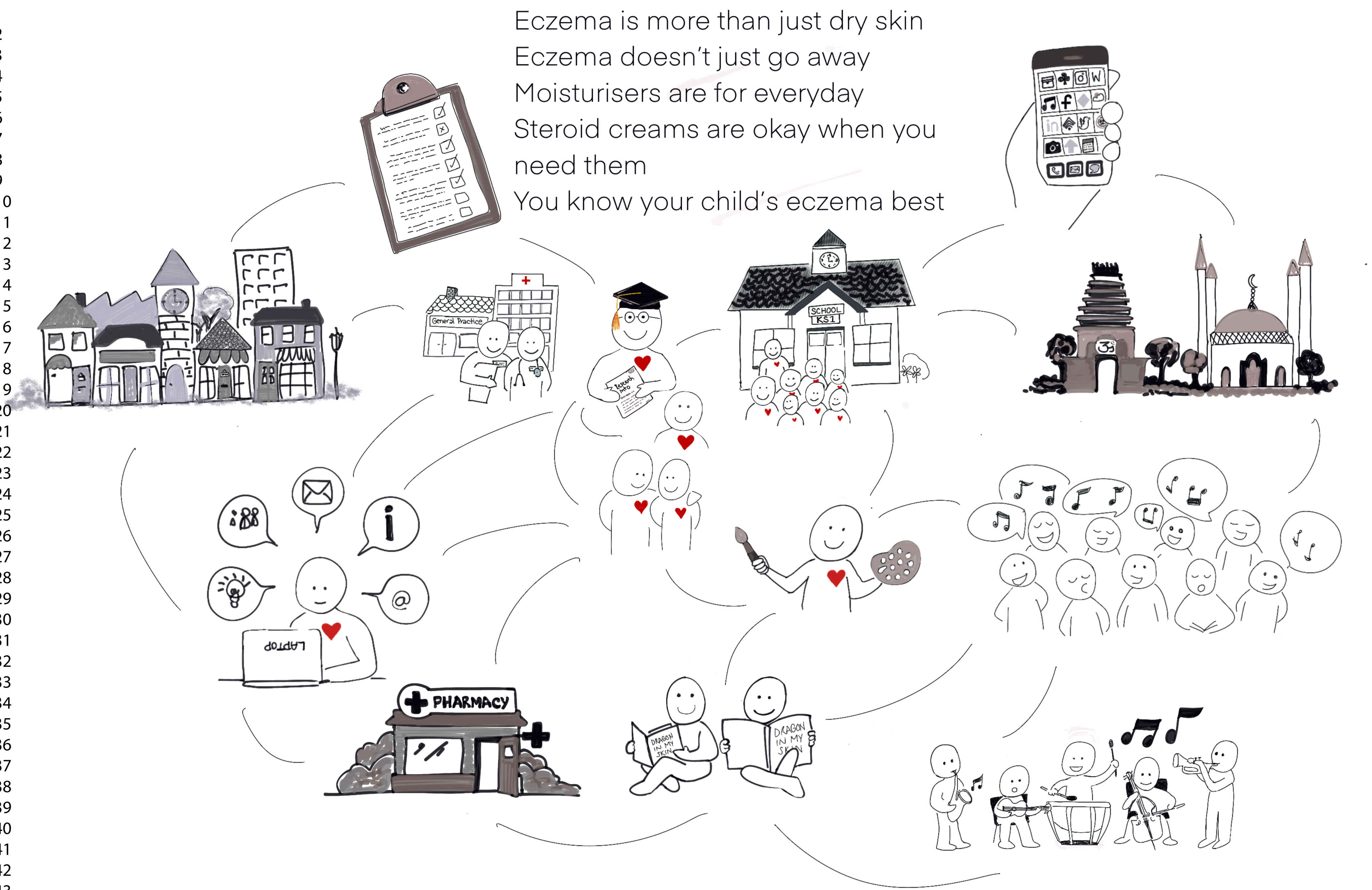
Eczema is more than just dry skin
Eczema doesn't just go away
Moisturisers are for everyday
Steroid creams are okay when you need them
You know your child's eczema best
General Practice
SCHOOL
KS1
PHARMACY
DRAGON IN MY SKIN
DRAGON IN MY SKIN

BMJ Open co-creation eczema mindlines COREQ 17.12.19

COREQ Statement

Knowledge mobilisation: An ethnographic study of the influence of practitioner mindlines on eczema self-management in primary care in the United Kingdom

**Statement**	**Page no**
**Domain 1: Research team and reflexivity**<br>*Personal Characteristics*<br>1. Interviewer/facilitator Which author/s conducted the interview or focus group?<br>FC was knowledge broker	5
2. Credentials What were the researcher's credentials? E.g. PhD, MD<br>DProf, RN	Title page
3. Occupation What was their occupation at the time of the study?<br>Professor of Nursing and Health Research	Title page
4. Gender Was the researcher male or female?<br>Female	Title page
5. Experience and training What experience or training did the researcher have?<br>I have extensive experience in qualitative research.	Title page
Relationship with participants<br>6. Relationship established Was a relationship established prior to study commencement?<br>**Knowledge broker**	Throughout manuscript
7. Participant knowledge of the interviewer<br>What did the participants know about the researcher? e.g. personal goals, reasons for doing the research	NA
8. Interviewer characteristics What characteristics were reported about the interviewer/facilitator? e.g. Bias, assumptions, reasons and interests in the research topic<br>Participants were aware, and it is reported in the manuscript, that I am a Registered Nurse with an interest in how eczema knowledge is developed and shared between patients and practitioners in primary care and that this was a publically funded study.	NA
**Domain 2: study design**<br>*Theoretical framework*<br>9. Methodological orientation and theory<br>What methodological orientation was stated to underpin the study? e.g. grounded theory, discourse analysis, ethnography, phenomenology, content analysis<br>Mindlines explained in background section	3-4
Participant selection<br>10. Sampling How were participants selected? e.g. purposive, convenience, consecutive, snowball<br>Purposive sampling to ensure a mix of co-creators	NA
11. Method of approach How were participants approached? e.g. face-to-face, telephone, mail, email	NA
12. Sample size How many participants were in the study?	NA
13. Non-participation How many people refused to participate or dropped out? Reasons?	NA
Setting<br>14. Setting of data collection Where was the data collected? e.g. home, clinic, workplace<br>Accessible conference centre and via email / telephone	8
15. Presence of non-participants Was anyone else present besides the participants and researchers?	NA
16. Description of sample What are the important characteristics of the sample? e.g. demographic data, date<br>Interview participants were sampled by profession, gender and years in practice.	Table 4
Data collection<br>17. Interview guide Were questions, prompts, guides provided by the authors? Was it pilot tested?	NA
18. Repeat interviews Were repeat interviews carried out? If yes, how many?	NA

For peer review only

BMJ Open co-creation eczema mindlines COREQ 17.12.19

19. Audio/visual recording Did the research use audio or visual recording to collect the data?	No
20. Field notes Were field notes made during and/or after the interview or focus group?<br>Notes and flip charts used	NA
21. Duration What was the duration of the interviews or focus group?	NA
22. Data saturation Was data saturation discussed?	NA
23. Transcripts returned Were transcripts returned to participants for comment and/or correction?<br>Data summaries were circulated to group members for comment, supplementation and modification	NA
**Domain 3: analysis and findings**<br>*Data analysis*<br>24. Number of data coders How many data coders coded the data?<br>Data analysis was completed by FC and RP	8
25. Description of the coding tree Did authors provide a description of the coding tree?<br>No	NA
26. Derivation of themes Were themes identified in advance or derived from the data?<br>Findings were derived from the data	8
27. Software What software, if applicable, was used to manage the data?<br>No	NA
28. Participant checking Did participants provide feedback on the findings?<br>Yes	NA
Reporting<br>29. Quotations presented Were participant quotations presented to illustrate the themes / findings? Was each quotation identified? e.g. participant number<br>Quotations are provided	8-15
30. Data and findings consistent Was there consistency between the data presented and the findings?<br>Yes.	8-15
31. Clarity of major themes Were major themes clearly presented in the findings?<br>Yes.	8-15
32. Clarity of minor themes Is there a description of diverse cases or discussion of minor themes?<br>Yes,	8-15

For peer review only

BMJ Open

Can co-created knowledge mobilisation interventions alter and enhance mindlines to improve childhood eczema care? A United Kingdom based Social Impact Framework evaluation.

Journal:	*BMJ Open*
Manuscript ID	bmjopen-2022-065557.R1
Article Type:	Original research
Date Submitted by the Author:	01-Mar-2023

Complete List of Authors:	Cowdell, Fiona; Birmingham City University Faculty of Health Education and Life Sciences, Faculty of Health Education and Life Sciences<br>Lax, Stephanie; University of Nottingham, Centre of Evidence Based Dermatology<br>Van Onselen, Julie; Independent Researcher<br>Pendleton, Rose ; Independent Researcher

**Primary Subject Heading**:	Evidence based practice
Secondary Subject Heading:	Dermatology
Keywords:	Eczema < DERMATOLOGY, QUALITATIVE RESEARCH, Paediatric dermatology < DERMATOLOGY

SCHOLARONE™
Manuscripts

I, the Submitting Author has the right to grant and does grant on behalf of all authors of the Work (as defined in the below author licence), an exclusive licence and/or a non-exclusive licence for contributions from authors who are: i) UK Crown employees; ii) where BMJ has agreed a CC-BY licence shall apply, and/or iii) in accordance with the terms applicable for US Federal Government officers or employees acting as part of their official duties; on a worldwide, perpetual, irrevocable, royalty-free basis to BMJ Publishing Group Ltd ("BMJ") its licensees and where the relevant Journal is co-owned by BMJ to the co-owners of the Journal, to publish the Work in this journal and any other BMJ products and to exploit all rights, as set out in our licence.

The Submitting Author accepts and understands that any supply made under these terms is made by BMJ to the Submitting Author unless you are acting as an employee on behalf of your employer or a postgraduate student of an affiliated institution which is paying any applicable article publishing charge ("APC") for Open Access articles. Where the Submitting Author wishes to make the Work available on an Open Access basis (and intends to pay the relevant APC), the terms of reuse of such Open Access shall be governed by a Creative Commons licence – details of these licences and which Creative Commons licence will apply to this Work are set out in our licence referred to above.

Other than as permitted in any relevant BMJ Author's Self Archiving Policies, I confirm this Work has not been accepted for publication elsewhere, is not being considered for publication elsewhere and does not duplicate material already published. I confirm all authors consent to publication of this Work and authorise the granting of this licence.

For peer review only

Article title: Can co-created knowledge mobilisation interventions alter and enhance mindlines to improve childhood eczema care? A United Kingdom based Social Impact Framework evaluation.

Corresponding author information:
Fiona Cowdell
DProf, RN
Professor of Nursing and Health Research
Faculty of Health, Education and Life Sciences
Birmingham City University
220 Ravensbury House
Westbourne Road
Edgbaston
Birmingham
B15 3TN
UK
Telephone: +44 (0)121 300 4345
Email: Fiona.cowdell@bcu.ac.uk
orcid.org/0000-0002-9355-8059

Stephanie Lax
PhD
Research Fellow
Centre of Evidence Based Dermatology
School of Medicine
Applied Health Research Building
University of Nottingham
NG7 2RD
Email: stephanie.lax@nottingham.ac.uk
orcid.org/0000-0002-7000-9364

Julie Van Onselen, RGN, RSCH, DipN, DipM, BA(Hons), Lecturer Practitioner in Dermatology, Dermatology Education Partnership Ltd and Nurse Adviser, National Eczema Society

Rose Pendleton
PPI Parent
c/o Birmingham City University

Word count: 4622

Number of figures: 3

Number of tables: 6

Keywords: atopic eczema, knowledge mobilisation, interventions, mindlines, contributions, social impact

For peer review only - http://bmjopen.bmj.com/site/about/guidelines.xhtml

Abstract

Title: Can co-created knowledge mobilisation interventions alter and enhance mindlines to improve childhood eczema care? A United Kingdom based Social Impact Framework evaluation.

Objective: To evaluate the impact of using knowledge mobilisation interventions to alter and enhance mindlines and improve childhood eczema care.

Design: The eczema mindlines study involved three stages: i) mapping and confirming eczema mindlines, ii) intervention development and delivery and iii) analysis of intervention impact. The focus of this paper is on stage three. Data analysis was guided by the Social Impact Framework to address the questions i) what is the impact of this study on individuals and groups? ii) what changes in behaviour and practice have occurred due to their involvement? iii) what mechanisms have enabled these impacts or changes to occur? and iv) what are the recommendations and questions arising from this research?

Settings: A deprived inner-city neighbourhood in central England, and national/international settings.

Participants: Patients, practitioners and wider community members exposed to the interventions locally, nationally and internationally.

Results: Data revealed tangible multi-level, relational and intellectual impacts. Mechanisms supporting impact included: simplicity and consistency of messages adapted to audience, flexibility, opportunism and perseverance, personal interconnectivity and acknowledgment of emotion. Co-created knowledge mobilisation strategies to alter and enhance mindlines mediated through knowledge brokering were effective in producing tangible changes in eczema care practice and self-management and in 'mainstreaming' childhood eczema in positive way across communities. These changes cannot be directly attributed to the KMb interventions however, the evidence points to the significant contribution made.

Conclusion: Co-created knowledge mobilisation interventions offer a valuable method of altering and enhancing eczema mindlines across lay-practitioner-wider society boundaries. The Social Impact Framework provides comprehensive method of understanding and documenting the complex web of impact occurring as a result of knowledge mobilisation. This approach is transferable to managing other long-term conditions.

For peer review only - http://bmjopen.bmj.com/site/about/guidelines.xhtml

Strengths and limitations

- New, methodical application of the Social Impact Framework in the context of knowledge mobilisation
- Robust approach to mapping how knowledge mobilisation interventions have altered and enhanced eczema mindlines
- The Social Impact Framework offers a comprehensive approach to assessing *contributions* to changes in practice or behaviour, but definitive *attribution* claims cannot be made

For peer review only - http://bmjopen.bmj.com/site/about/guidelines.xhtml

Can co-created knowledge mobilisation interventions alter and enhance mindlines to improve childhood eczema care? A United Kingdom based Social Impact Framework evaluation.

Introduction

Co-created knowledge mobilisation (KMb) interventions have the potential to influence the stubborn evidence-practice gap in healthcare but measuring impact of these approaches is challenging. Childhood atopic eczema (AE) is a common and bothersome skin condition (1) which requires regular and ongoing self-management (2). AE is predominantly treated in primary care (3) and a robust evidence base for treatment exists (4, 5). Effective self-management requires a level of shared knowledge, language and understanding between patient and practitioner (6).

Co-methodologies in healthcare are widely considered to be a 'good thing' although the language of 'co' working is not fully defined and remains a fundamentally contested concept (7). The terms co-design, co-production, co-creation, participatory research or participatory design are progressively used, sometimes interchangeably by researchers (8) and research funders (9). Regardless of this, co-methodological working is gaining traction in healthcare (10) and it is widely acknowledged that research engaging end-users is more likely to have an impact on practice (11).

KMb interventions are increasingly used in healthcare to address multiple gaps between evidence, knowledge and action (12). It requires purposeful efforts to create, disseminate and operationalise knowledge from multiple sources (13). KMb is context specific (14), relational (15) and socially constructed (16). It is a rapidly evolving and wide-ranging field; currently there are in excess of 47 models (17) and 71 published reviews (18) and a Google search yields 72,800,000 hits. Selecting approaches to KMb can be problematic with some being highly theoretical and difficult to apply in practice. One pragmatic approach which is firmly embedded in day-to-day practice is alteration and enhancement of 'mindlines'. Mindlines are 'collectively reinforced, internalised tacit guidelines' which underpin clinical decision making (19), particular emphasis is on contextual relevance and application of knowledge. Mindlines are developed from multiple knowledge sources such as communication with colleagues and opinion leaders and from personal tactic knowledge developed over time, knowledge is socially transmitted in the context of its use (19). Mindlines build on the work of Nonaka and colleagues (20) who propose the **S**ocialisation, **E**xternalisation, **C**ombination, **I**nternalisation (SECI) spiral to guide implementation of new knowledge into practice. The SECI spiral comprises, socialisation (surfacing tacit knowledge through shared experiences), externalisation (articulating tacit knowledge into explicit knowledge), combination (combining exposed explicit knowledge with more complex and systematic explicit knowledge, for example clinical guidelines, to

For peer review only - http://bmjopen.bmj.com/site/about/guidelines.xhtml

develop new knowledge) and internalisation (embodying this new knowledge as tacit knowledge for day-to-day use).

Impact of KMb is notoriously hard to measure. To date the focus in healthcare has primarily been on moving new knowledge to clinicians and policy makers, with less attention paid to KMb across communities (21, 22). Effective evaluation of KMb activity is essential to better understand if and how stakeholders across communities use new knowledge and to refine strategies (23). The Social Impact Framework, although primarily directed to evaluating co-production offers a comprehensive and structured approach to understanding and documenting micro-meso-macro levels, processes, impacts and mechanisms of the KMb activity and to map the winding pathway of incremental and often subtle changes which are readily overlooked (24). Beckett et al (12) provide a worked example of application of the SIF and their suggested questions are used here to guide analysis (Table 1):

1. What is the impact of this study on individuals and groups? (Outcome 1)
2. What changes in thinking, behaviour and practice have occurred due to their involvement? (Outcome 2)
3. What mechanisms have enabled these impacts or changes to occur? (Outcome 3)
4. What are the recommendations and questions arising from this research? (Outcome 4).

Table 1: Social Impact Review questions (from Beckett et al (12))

Method

The eczema mindlines study involved three phases: **Stage 1:** Mapping and confirming eczema mindlines, **Stage 2:** Intervention development and delivery and **Stage 3:** Analysis of intervention impact (see Figure 1). The focus of this paper is on Phase 3, for context and clarity summaries of Phases 1 & 2 are included.

Insert Figure 1: Evidence sources, stages and outcome measures (adapted from Beckett et al (12))

Phase 1: Mapping and confirming eczema mindlines

Phase 1 comprised two elements. Firstly, an ethnographic study to map lay and practitioner eczema mindlines in one deprived inner-city area in the UK (25, 26). Secondly an interview study with a

For peer review only - http://bmjopen.bmj.com/site/about/guidelines.xhtml

wider population to confirm and expand understanding of lay and practitioner eczema mindlines (27).

Phase 2: Intervention development and delivery

In a series of co-creation workshops involving people living with eczema, practitioners and researchers combined their tacit knowledge and data from phase 1 with existing research evidence. Co-creators concluded that to alter and enhance existing eczema mindlines five key, consistent, evidence-based messages needed to be shared (Table 2).

i)	Eczema is more than just dry skin
ii)	Eczema doesn't just go away
iii)	Moisturisers are for every day
iv)	Steroid creams are okay when you need them
v)	You know your child's eczema best

Table 2: Five co-created eczema messages

Crucially these messages needed to be transmitted across lay-practitioner-wider society boundaries using a range of techniques to enhance shared knowledge, understanding and language. For lay co-creators 'trust' and 'realness' of messages was important and HCPs wanted practical, locally relevant, hints and tips, tailored, 'no faff' approaches (28). KMb interventions were developed in light of the characteristics of evidence and context in order to determine the best approaches (29).

Intervention delivery was grounded in four schools of thought which cumulatively ensured knowledge was mobilised in the right format for the right audience and effectively spread across boundaries as summarised in Table 3.

Knowledge brokering

- Knowledge brokers build networks and facilitate opportunities to share knowledge (30). This was the core of all KMb activity.

'Ba'

- 'Ba' is a shared space for knowledge generation and spreading (31-33) which aligns with the Socialisation, Externalisation, Combination, Internalisation spiral from which mindlines evolved. In this case the Ba space was the locality of the original research, a deprived inner-city catchment. The intention was to achieve a density of KMb in a local

For peer review only - http://bmjopen.bmj.com/site/about/guidelines.xhtml

area to support shared understandings and language across lay-practitioner-wider society boundaries.

Ripple effect model

- Ripple effect was used to amplify the impact of each KMb action, so one event produces effects which spread and produce further effects (34, 35).

Social marketing

- Social marketing goes beyond simply conveying knowledge widely and is intended to directly influence health care actions (36). Here emphasis was on community outreach (37).

Table 3: Underpinning approaches to KMb

Using the strategies outlined above the five co-created messages were shared using multiple interventions as summarised in Table 4 and Figure 2. The role of the knowledge broker, initially FC, and later a wider group of people involved in the KMb interventions (for example teachers) was pivotal. It involved working collaboratively with key stakeholders to enable transfer and exchange of knowledge across boundaries in different contexts (38) and using varied mechanisms (as discussed in Outcome 3). Messages were integrated into a children's book 'The Dragon in My Skin' (hereafter Dragon) with associated animation, song and teacher resources. Dragon resources were endorsed by the National Eczema Society and the Royal College of Nursing to enhance confidence the content was real and trustworthy as required by lay co-creators.

**KMb materials**	**Recipients**
Postcards and posters with key messages and supplementary information (Figure 2)	HCPs in local area including GPs, GP trainees, practice nurses, health visitors, community pharmacists and pharmacy counter assistants<br><br>Displayed in local infant and primary schools, libraries, places of worship, GP practices, community pharmacies
Mindline informed educational sessions, led by DNS	Health visitors, community public health nurses (n=36), GPs (n=18), practice nurses (n=8)
Shopping centre pod for rapid consultations with two DNSs	Consultations with customers (n=94)
In person and online story reading and activity sessions in<br>• nurseries and primary schools<br>• places of worship	Children (n=86), teachers and teaching assistants (n=11)<br>Children and parents (n=~50)
Eczema mindlines website	Freely available online
The Dragon in My Skin book the-dragon-in-my-skin-132634726304040297.pdf (windows.net)	Freely available online

For peer review only - http://bmjopen.bmj.com/site/about/guidelines.xhtml

	Hard copies distributed to primary schools (n=792) with links to all other Dragon resources
Dragon workshops	Children with eczema (n=10), their parents, an author and professional orchestra members
Dragon premiere	Children with eczema, their parents, professional orchestra members and invited guests with an interest in eczema including practitioners and members of eczema organisations (n=62)
Dragon book the-dragon-in-my-skin-132634726304040297.pdf (windows.net)	Freely available online<br>Hard copies distributed to primary schools (n=792)
Dragon Teacher resource pack tdims-workpack-v2-long-132693393982395364.pdf (windows.net)	Freely available online
Dragon Animation The Dragon In My Skin - School of Health Sciences | Birmingham City University (bcu.ac.uk)	Freely available online
Dragon translations	Will be freely available online
Eczema mindlines documentary https://youtu.be/C4d_yxvHVPk	Freely available online

Table 4: KMb interventions

Insert Figure 2: Example of postcards with key messages and supplementary information

Stage 3: Analysis of intervention impact

Aim

To systematically evaluate the impact of co-created KMb interventions in altering and enhancing mindlines and improving childhood eczema care.

Design

Social Impact Framework evaluation

Data collection

Data collection was multi-factorial, data sources and collection methods are summarised in Table 5.

**Data source**	**Data collection method**
HCPs including GPs, GP trainees, practice nurses, health visitors, community pharmacists and pharmacy counter assistants	Qualitative interviews, informal conversations, email feedback
Health visitor and community practitioners	Post session evaluation and subsequent email feedback
Children, parents and artists engaged in Dragon work	Observation, informal conversations, email feedback
Attendees at world premiere of Dragon event	Immediate comments, Zoom chat, follow-up emails

For peer review only - http://bmjopen.bmj.com/site/about/guidelines.xhtml

Teachers and student teachers	Online survey pre and post using Dragon resources in practice, informal conversations
Charitable organisations	Email, informal conversations
Professional organisations	Email feedback, testimonial
Social media	Metrics and testimonials
Researcher	Reflective diary of observation and experiences at all stages

Table 5: Summary of data sources and data collection methods

Data analysis

Data analysis was guided by the Social Impact Framework (SIF) (12, 24) to capture multi-level processes, impacts and key mechanisms of our KMb activities. We collated data from all sources including transcripts of audio recorded interviews, feedback from online meetings and events, testimonials, email correspondence, researcher observation and conversations, online surveys and metrics. Many artefacts such as children's drawings were sent to us with a written description of the thought and emotion behind them from either the child or the teacher. Where no words were offered we described pictures in words to try to capture their essence. The varied data could not and should not be separated from knowledge of the study design and the condition and creation of data (39). The language and 'things' were inseparable (40), and so were analysed together using the collated datasets. Two authors (FC and RP) iteratively read, thought and wrote about and discussed the data in its entirety. We then interrogated data to address the four social impact review questions (Table 1) (41). Evaluation is reported according to the four outcomes around: impact on individuals and groups, changes in thinking, behaviour and practice, mechanisms enabling these impacts and finally recommendations and questions.

Reflexivity

A reflexive stance was maintained for the duration of the study acknowledging both the complexities of the world and researcher entanglement with the fullness of the research process (42) and our preconceived understandings.

Patient and Public Involvement

Lay people were involved in the development of the research question. They co-created the five key messages in a series of workshops, contributed to KMb planning and delivery and one representative is a co-author of this paper. All PPI activity was conducted in line with National Guidance (43).

Results

For peer review only - http://bmjopen.bmj.com/site/about/guidelines.xhtml

Results are documented according to the four outcomes.

Outcome 1: What is the impact of this study on individuals and groups?

Impact on individuals and groups was significant. Members of the co-creation group (n=22 lay people, practitioners and researcher) reported new understandings of eczema care from the 'other' perspective, *"conversations show how little lay people and HCPs understand each other's worlds and how interested they are in getting new insights" [researcher].* Participants demonstrated a new respect for the skills, knowledge and experiences of others and similarly gained a deeper understanding of the challenges and constraints of others. Co-creation enabled cross-fertilisation of ideas alongside a realisation of the power each person has to make a difference. Lay members found new ways of, and confidence in, communicating with practitioners and researchers.

At the time of writing engagement with the mindlines webpage, Facebook and Twitter is modest particularly given the vast potential audience numbers (Table 6); this suggests that personal approaches may be more valuable.

- All time views on the mindlines webpage to date 1146, split between documentary (n=386), animation (n=696) and knowledge nuggets (n=54).
- Direct views to the video guides n=121. View quality was high, people spent on average of five minutes across all pages.
- Uptake on Facebook and Twitter achieved nine and 121 followers respectively.
- Animation views for the five messages were: i) eczema doesn't just go away (n=270), ii) eczema is more than just dry skin (n=684), iii) moisturisers are for every day (n=58), iv) steroid creams are okay when you need them (n=223) and v) you know your child's eczema best (n=23).

Table 6: Engagement with mindlines website

Strategically placed posters and postcards impacted on the thinking of staff. Many either had or knew others who had eczema and thought the resources would be helpful. Pharmacy counter assistants supported adding a pack of postcards with all dispensed eczema topical treatments. A shopping centre pod set up for rapid consultations with two Dermatology Nurse Specialists (DNSs)

For peer review only - http://bmjopen.bmj.com/site/about/guidelines.xhtml

attracted 94 people in one day. Immediacy, advice from an expert and personalisation of the five key messages was highly valued in verbal feedback. It provided a basis for individuals to change their eczema self-management (although it is not possible to know whether this change was enacted). Children engaged enthusiastically with story reading and activity sessions Teachers reported ongoing conversations about how it must feel to live with eczema and increased empathy both themselves and amongst children. *One teacher reported "we had a lot of discussion around the topic of eczema and talked about feelings and how things such as eczema can affect our moods".* Attendance at mindline informed eczema sessions for HCPs was higher than anticipated. Evaluation was overwhelmingly positive, particularly in terms of contextual relevance and applicability to own practice, for example "it *stuck in my mind, direct relation to patients, the "hook" to apply to own practice* [GP].

The Dragon book was described as "*beautifully profound in its simplicity*" [orchestra member] and "*superb, I love the story and the pictures, what a lovely way for children with eczema to be able to see how they can tame their dragon and to have its impact validated in such a wonderful way!"* [lay person]. Through word-of-mouth connections around 200 further books were sent to other educators and HCPs. A Dermatology Specialist Nurse shared the book with children attending her clinic and wrote "*They love it, the children feel they have more control and it have made them feel special ………. this for me has been one of the best tools to use*".

Children enjoyed the Dragon online co-creation sessions "[child's name] *always looked forward to her sessions on zoom and you all made it so easy to engage and be confident. It's almost a shame the sessions are finished!*". Parents commented "*You guys do a great job at engaging the kids …… because you're after their input they're invested early on*" and also valued validation of the realities of living with eczema. They were proud to be part of the online premiere. Feedback from this included

- *"emotional…… this really helped me see what [my child] is feeling" [parent].*
- One mother described her daughter's anger at having to manage the condition was struck *"to hear that [anger] validated in a book for [my child] to understand"*
- *"It's beautiful. And as someone with a dragon since day one in life and struggling at the moment with it, I was especially moved by this." [teacher]*
- *"So often it's seen as 'just eczema'... it's nice to have something that shows how hard it is"* [parent],

For peer review only - http://bmjopen.bmj.com/site/about/guidelines.xhtml

- *"loved the way you haven't shied away from the difficult and painful experiences and feelings children have about eczema"* [charitable organisation]
- *"you've taken a debilitating but common and overlooked problem and made it come alive … I found it very moving"* [HCP].

Parents noted the benefits of greater awareness of eczema amongst teachers and other children through widespread sharing of the Dragon resources *"I am so glad that this will now be shared in schools to raise awareness amongst children of what some of their friends are going through"* [parent], *"Just awesome. That's so good. I'll send it to [child's name] teacher because there's a little girl in his class really suffering" [parent].* The YouTube animation has been viewed 1,378 times with 61.2% of views outside the United Kingdom, the average view duration was 3 minutes and 29 seconds and 22% of viewers watched all content relating to the five key messages (up to 9mins 45secs).

Outcome 2: What changes in behaviour, practice and research have occurred due to their involvement?

Children and parents

Tangible changes in behaviour and practice were described. Some children and parents recounted more concordance with treatment, for example "[child's name] *wanted me to tell you that she has put her spray on all over to look after her dragon"* [parent]. However, for most the more important change was the recognition that others gave to their child's eczema "*so often it's seen as 'just eczema' ... it's nice to have something that shows how hard it is*" [parent].

Health care practitioners

Practitioners reported not necessarily learning anything new but rather 'fine-tuning' their mindlines and changing in thinking for example Dragon "*validates experiences and feelings …… shows we understand*" [HCP]. Examples of simple but effective practice changes included:

- *"the three main things that I took away from it were using one application of steroids is just as good as two. Go big early with the steroid and go greasier as well, really, rather than have a kind of hierarchy, going for a greasier emollient earlier rather than wait"* [nurse] several months post-intervention the same nurse reported "*it's certainly made me more confident in prescribing,*

For peer review only - http://bmjopen.bmj.com/site/about/guidelines.xhtml

really. I don't think patients are coming back as much, I think actually going bigger earlier has a positive effect, really"

- *"I think probably we've often a bit mean with it …. I've double checked that they've got enough of the emollients"* [GP].
- *"I readily use the information on my contacts at home visits and during clinic times, it is valuable to my practice and aids my prescribing for children with skin conditions, and when to refer"* [HV].
- *"I use [postcards] in practice and in teaching my students about care of the skin on a regular basis"* [HV].

Teachers

Use of resources led to more understanding from teachers and peers, "*used it [book] with her individually to help the child manage her emotions and consider how she could manage her condition in school and that now the little girl picks the book up to read whenever she needs a 'comfort blanket' moment*" [teacher]. Teachers using Dragon resources conveyed value through words and images. "*The children had some really mature discussion during this lesson and I have to say I was impressed, a couple of children with eczema were heavily involved in this and told other pupils some of their experiences (without being prompted or pressured to do so)" [teacher]. "I have looked at this story with children in my class and they absolutely loved it. I created a hook where I had an animated dragon that came into our classroom and left some footprints and burnt paper and it got the children wondering why we had a dragon come in. The children then created their own story maps and understood the concept of eczema, as we have two girls who suffer from it. It was amazing*", see Image 1. The implication from teacher feedback was that other children developed greater empathy for peers with eczema with the suggestion that this approach would reduce unkindness and bullying.

Outcome 3: What mechanisms have enabled these impacts or changes to occur?

Multiple mechanisms enabled impact including: simplicity and consistency of messages adapted to audience, flexibility, opportunism and perseverance, personal interconnectivity and acknowledgment of emotion.

Simplicity and consistency of messages adapted to audience

All KMb was underpinned by the five simple, key messages. Although not new these messages are at the heart of most eczema care with the mantra being "get control-keep control" through use of

For peer review only - http://bmjopen.bmj.com/site/about/guidelines.xhtml

topical corticosteroids when needed and regular and consistent application of emollients. Consistent, cross boundary messaging was intended to bring about shared language and understanding on which to base more equal eczema consultations. The role of the DNSs was pivotal. Mindline informed teaching engaged HCPs, using their own stories to *"hang things on"* allowing immediate contextualisation and application of new knowledge. Equally having an expert with a wealth of current clinical and research knowledge and a repertoire of anecdotes made session rich, relevant and real. Lay people relished the opportunity to get on-the-spot expert, personal advice at the shopping centre. Immediacy was key to success. The five key messages provided a scaffold for each consultation, essentially each person received the same key information, but the DNSs, trusted sources of information, skilfully adapted and integrated messages to make them meaningful and useful to each individual.

The Dragon offered the five messages in child-friendly formats which addressed eczema care from a positive, proactive standpoint rather than the more usual problematisation of the condition. Dragon related KMb activity has grown exponentially mainly by word of mouth supplemented by sharing in newsletters, magazines and websites. Numerous requests for resources have been received from HCPs and teachers. For example, one National organisation with a mission to transform localities with creativity and culture wrote the Dragon is "*Beautifully composed, created, animated and such a positive piece for children and young people to be involved in when eczema can be so hard"* and went on share the resources across wide-ranging networks. An attendee at the Dragon premiere descried the resources as "*incredible"* and shared them with every primary school and primary care practice in one region. An education leader who heard about the Dragon through a personal contact wrote "*I am delighted to be able to share these resources with our 87 mental health leads …. as I believe that this resource can support reducing the stigma linked to eczema, often born out of ignorance of the condition".* Through HCP contacts Dragon resources are in the process of being translated and culturally adapted into French and Portuguese.

Flexibility, opportunism and perseverance

Diligent, persistent, adaptable and proactive knowledge brokering was an essential element of enabling impact, as was perseverance in the face of practical and process constraints. Perseverance and patience were required in managing bureaucracy in setting up events and when events were cancelled at the last minute and needed to be rebooked. Some people rejected my offer outright including one children's play venue manager who would not support anything that suggested steroid creams were okay when you need them and a leisure centre manager who stated the messages

For peer review only - http://bmjopen.bmj.com/site/about/guidelines.xhtml

were *"not suitable"*. Effective knowledge brokering also relied on i) building robust and enduring relationships with leading eczema charities and professional organisations and securing their endorsement ii) engaging with influencers, authority figures and decision-makers and iii) openness to collaborative working across new networks. In the first instance FC was the sole knowledge broker but over time others took on this role in different contexts (for example teachers shared messages and resources far and wide), thus building up a knowledge sharing network.

Personal interconnectivity

Personal interconnectivity was a key factor in sharing messages (Figure 3). Through personal contacts, email and telephone calls multiple individuals and organisations were contacted offering to share the key eczema messages using approaches tailored to each venue. Personal networks were effective door openers, for example a practice manager introduced me to an Imam, who introduced me to a pharmacist, and so the ripple went on, allowing me to access many groups I would not otherwise have reached. Equally it was the starting point and central in developing, sharing and using Dragon resources. The idea was influenced by conversations with a patient group who highlighted the need to make teachers and children more aware of eczema and mainstream it rather than using existing problematising approaches such as having a special assembly on the condition. A chance conversation with a colleague led to development of the Dragon teacher resource pack. Consideration was given to the limitations of personal connections. We all inherently move in our own limited circles however, we strived for inclusivity through situating our work 'out there' and using the ripple effect to meet new and unexpected allies.

Insert Figure 3: Illustration of interconnectivity

Acknowledgment of emotion

Tapping into emotions amplified the impact of KMb activity on altering and enhancing mindlines. For HCPs relating knowledge to individual patients and their families was more powerful than generic teaching and sessions also gave space to express the frustrations of eczema care and collaboratively seek more positive approaches. For lay consultations being "*listened to*" as a whole person was key. Numerous Dragon comments focused on emotion as much as content, for example a teacher wrote "*It is such a wonderful concept that will make such a difference to children with and without eczema. I know me and my daughter would have felt much happier at school if we'd had something like this"*. An experienced HCP commented "*You've taken a debilitating but common and overlooked problem and made it come alive! I loved it all and found it very moving"* and many parents echo the

For peer review only - http://bmjopen.bmj.com/site/about/guidelines.xhtml

sentiment of a charity leader "*Loved the way you haven't shied away from the difficult and painful experiences and feelings children have about eczema*".

Outcome 4: What are the recommendations and questions arising from this research?

This research has important implications in terms of future KMb activity.

- Firstly, altering and enhancing mindlines across patient-practitioner-wider society boundaries is possible and effective in changing behaviour / practice. Mindlines inherently made sense to all participants. Existing evidence was used to inform development of key, simple messages that were shared using creative and contextually adroit (19) formats that were relevant and applicable for end users.
- Secondly, knowledge brokering may start with one person but building up networks of knowledge brokers is essential. In this instance the process was organic and was strengthened by openness to unexpected opportunities. In future thought must be given to potential networks but equally researchers need to be open to and actively seeking new possibilities.
- Finally, the Social Impact Framework offers a robust and iterative approach to planning, mapping and evidencing impact. 'Proving' the value of KMb is not and never will be straightforward. However, adoption of the SIF offers a step-change in demonstrating wide-ranging impact of KMb activity.

Discussion

The aim of this study was to evaluate the impact of using co-created KMb interventions to alter and enhance mindlines and improve childhood eczema care. It is one of the first to methodically evaluate the impact of using KMb interventions to alter and enhance mindlines across patient-practitioner-wider society boundaries. The evidence presented demonstrates the resonance that the work as a whole had with people living eczema and those providing care. Recognition of the challenges and use of contextually relevant interventions for both appear to have increased receptivity and integration of new knowledge into everyday care. We are confident that eczema mindlines have been altered and enhanced. We have demonstrated that the SIF, which has a sound theoretical base, offers an effective and comprehensive approach to evaluating impact of KMb interventions. Use of the SIF has enabled reflection on the complex web of impact from a range of perspectives which may be overlooked if using more traditional measures. We are mindful that this work has limitations. We have made a *contribution* to changes in practice or behaviour but cannot definitively

For peer review only - http://bmjopen.bmj.com/site/about/guidelines.xhtml

attribute this change to our interventions. However, the evidence presented suggests changes in people's thinking which is likely to influence their actions. Reporting is in accordance with the consolidated criteria for reporting qualitative research (44).

Methodical assessment of the impact of KMb, activity is scarce (45, 46) despite allied literature pointing to the need to build understanding (47) and competence (48) in this arena. Alternative approaches to evaluating KMb are available for example The Community Knowledge Mobilization Framework (49) however this is more limited than the SIF particularly in terms of considering breadth and mechanisms of change. Impact is a contested term, sometimes conceptualised as a linear process (50) in which impact is *directly attributable* to generation and dissemination of new knowledge (51). In the present study impact was viewed from the wide-ranging lens of the SIF. We are mindful that there are many other influences on eczema care and that this work offers a *contribution* to change (52). Application of the SIF has allowed a nuanced understanding of the depth and breadth of impact of KMb activities and contributed to the much-needed development of KMb theory (46). The SIF although primarily directed to evaluating co-production, offered a structured approach to reflect on micro-macro levels, processes, impacts and mechanisms of the KMb activity and map the winding pathway of incremental and often subtle changes which are readily overlooked.

The KMb interventions used to share simple consistent messages, co-created by end users is congruent with current thinking about challenges of KMb. Extant literature points to i) information overload for HCPs (53) and lay people (54), ii) inconsistent advice regarding eczema care (55), iii) poor quality information and limited confidence in assessing veracity of available information for lay people (56), iv) the need to consistently work with end users to increase uptake of knowledge (57) and v) the value of promoting shared language and understandings and thus support shared decision making and self-management (58). Gabbay and Le May (19) identify the inter-relationship of patient-practitioner mindlines and hence the need to change mindlines in parallel. However, few studies have considered KMb across lay-practitioner-wider society boundaries (21).

Knowledge brokers as intermediaries between researchers and practitioners are well established in healthcare as evidenced in recent reviews (59, 60). Nevertheless, the role can be problematic with some brokers challenged by role ambiguity and the need for a multidimensional skill set (61). In the present study the broker being a researcher and nurse and having lived experience of eczema minimised these tensions and were of distinct benefit in the relationship brokering component of

For peer review only - http://bmjopen.bmj.com/site/about/guidelines.xhtml

the role (62). Over time others took up brokering activity, which enhanced capacity to move evidence to practice (61).

Systematic analysis of KMb activity has highlighted multiple mechanisms influencing impact which may be applied in future KMb work. In the present study key processes included: i) engagement of key stakeholders and end users, ii) appreciative engagement, creating opportunities for engagement, valuing unique individual contributions and respectful working, iii) diligent, persistent and proactive knowledge brokering, iv) sustained supportive relationships, v) use of iterative flexible processes, adjustment to contextual challenges and changing circumstances and vi) creativity and use of diverse media. The KMb materials provide lay people HCPs and teachers with evidence-based resources to use and share with others. We also offer a novel approach to systematically evaluating KMb activity which builds much needed theory alongside practical application. There is still much work to be done to better understand the impact of knowledge mobilisation strategies specifically those striving to bridge lay-practitioner-wider society boundaries to improve care.

Conclusion

This study is one of the first to systematically assess the impact of KMb interventions designed to alter and enhance mindlines across lay-practitioner-wider society boundaries. The Social Impact Framework has been used to transparently map the complex web of impact from a range of perspectives which may be overlooked if using more traditional measures. Crucially impact has included tangible changes in childhood eczema care practice and self-management and ‘mainstreamed’ the condition to enhance understanding of children and teachers. It brings to the fore new understandings of key mechanisms underpinning effective KMb practice. The challenge now is to test this approach to assess the impact of other types of KMb interventions.

For peer review only - http://bmjopen.bmj.com/site/about/guidelines.xhtml

Contributionship statement

FC: Led the research, including study design, data acquisition and interpretation and writing this article. She is accountable for all aspects of the work in ensuring that questions related to the accuracy or integrity of any part of the work are appropriately investigated and resolved.

SL: Contributed to study design, critical revision and gave approval for submission

RP: Contributed to study design, data acquisition and interpretation, critical revision and gave approval for submission

JVO: Contributed to data acquisition, critical revision and gave approval for submission

Competing interests

The authors have no competing interests to declare

Funding

This report is independent research arising from a Knowledge Mobilisation Research Fellowship, Professor Fiona Cowdell, KMRF-2015-04-004 supported by the National Institute for Health Research. The views expressed in this publication are those of the authors and not necessarily those of the NHS, the National Institute for Health Research, Health Education England or the Department of Health.

Data sharing statement

Data are available upon reasonable request to the corresponding author

Statement of Ethics Approval

This manuscript presents evaluation data only and so does not require ethics approval.

Acknowledgments

We extend our thanks to all who have taken part in this study and to Kate Beckett who has kindly shared her knowledge and expertise when commenting on drafts of this manuscript. Thanks also to Jay Nolan Latchford and Michaela Tait for creating images.

For peer review only

References

1. Abuabara K, Ye M, McCulloch CE, Sullivan A, Margolis DJ, Strachan DP, Paternoster L, Yew YW, Williams HC, Langan SM. Clinical onset of atopic eczema: results from 2 nationally representative British birth cohorts followed through midlife. Journal of Allergy and Clinical Immunology. 2019 Sep 1;144(3):710-9.
2. Teasdale E, Muller I, Sivyer K, Ghio D, Greenwell K, Wilczynska S, Roberts A, Ridd MJ, Francis N, Yardley L, Thomas KS. Views and experiences of managing eczema: systematic review and thematic synthesis of qualitative studies. British Journal of Dermatology. 2021 Apr;184(4):627-37.
3. Le Roux E, Powell K, Banks JP, Ridd MJ. GPs' experiences of diagnosing and managing childhood eczema: a qualitative study in primary care. British Journal of General Practice. 2018 Feb 1;68(667):e73-80.
4. National Institute for Heath and Care Excellence. Atopic eczema in under 12s: diagnosis and management. Clinical guideline (CG57) updated March 2021 Overview | Atopic eczema in under 12s: diagnosis and management | Guidance | NICE Accessed 24.01.22
5. National Institute for Heath and Care Excellence, Clinical Knowledge Summary, Eczema-atopic Eczema - atopic | Health topics A to Z | CKS | NICE Accessed 24.01.22
6. Ellis J, Boger E, Latter S, Kennedy A, Jones F, Foster C, Demain S. Conceptualisation of the 'good'self-manager: A qualitative investigation of stakeholder views on the self-management of long-term health conditions. Social Science & Medicine. 2017 Mar 1;176:25-33.
7. Williams O, Sarre S, Papoulias SC, Knowles S, Robert G, Beresford P, Rose D, Carr S, Kaur M, Palmer VJ. Lost in the shadows: reflections on the dark side of co-production. Health Research Policy and Systems. 2020 Dec;18:1-0.
8. Greenhalgh T, Papoutsi C. (2018) Studying complexity in health services research: desperately seeking an overdue paradigm shift. *BMC Med.* 16 (95) doi:10.1186/s12916-018-1089-4
9. INVOLVE Guidance on co-producing a research project 2018 Copro_Guidance_Feb19.pdf (invo.org.uk) Accessed 24.01.22
10. Cowdell F, Dyson J, Sykes M, Dam R, Pendleton R. How and how well have older people been engaged in healthcare intervention design, development or delivery using co-methodologies: A scoping review with narrative summary. Health & Social Care in the Community. 2020 Oct 25.
11. Greenhalgh T, Jackson C, Shaw S, Janamian T. Achieving research impact through co-creation in community-based health services: literature review and case study. The Milbank Quarterly. 2016 Jun;94(2):392-429.
12. Beckett K, Deave T, McBride T, le May Andrée A, Gabbay J, Kapoulas U, Long A, Warburton G, Wogan C, Cox L, Thompson J. Using Forum Theatre to mobilise knowledge and improve NHS care: The Enhancing Post-injury psychological Intervention and Care (EPPIC) study. Evidence and Policy. https://doi.org/10.1332/174426421X16420902769508
13. Haynes A, Rowbotham S, Grunseit A, Bohn-Goldbaum E, Slaytor E, Wilson A, Lee K, Davidson S, Wutzke S. Knowledge mobilisation in practice: An evaluation of the Australian Prevention Partnership Centre. Health research policy and systems. 2020 Dec;18(1):1-7.
14. Kislov R, Waterman H, Harvey G, Boaden R. Rethinking capacity building for knowledge mobilisation: developing multilevel capabilities in healthcare organisations. Implementation Science. 2014 Dec;9(1):1-2.
15. Powell A, Davies H, Nutley S. Missing in action? The role of the knowledge mobilisation literature in developing knowledge mobilisation practices. Evidence & Policy: A Journal of Research, Debate and Practice. 2017 13(2):201-23.

For peer review only - http://bmjopen.bmj.com/site/about/guidelines.xhtml

16. Marshall M, Eyre L, Lalani M, Khan S, Mann S, de Silva D, Shapiro J. Increasing the impact of health services research on service improvement: the researcher-in-residence model. Journal of the Royal Society of Medicine. 2016 Jun;109(6):220-5.
17. Ward V. Why, whose, what and how? A framework for knowledge mobilisers. Evidence & Policy. 2017 13(3):477-497 . https://doi.org/10.1332/174426416X14634763278725
18. Davies HTO, Powell AE, Nutley SM. Mobilising knowledge to improve UK health care: learning from other countries and other sectors – a multimethod mapping study. NIHR Journals Library, Southampton (UK); 2015. PMID: 26110190.
19. Gabbay J, Le May A. Practice-based evidence for healthcare: clinical mindlines. Abingdon, Routledge; 2011.
20. Nonaka I, Toyama R, Konno N. SECI, Ba and leadership: a unified model of dynamic knowledge creation. Long range planning. 2000 Feb 1;33(1):5-34.
21. Appleby B, Cowdell F, Booth A. Knowledge mobilization in bridging patient-practitioner-researcher boundaries: A systematic integrative review. Journal of Advanced Nursing. 2021 Feb;77(2):523-36.
22. Weiss D, Lillefjell M, Magnus E. Facilitators for the development and implementation of health promoting policy and programs–a scoping review at the local community level. BMC Public Health. 2016 16(1):1-5.
23. Worton S, Loomis C, Pancer S, Nelson G, Peters RD. Evidence to impact: A community knowledge mobilisation evaluation framework. Gateways: International Journal of Community Research and Engagement. 2017 10:121-42.
24. Beckett K, Farr M, Kothari A, Wye L, Le May A. Embracing complexity and uncertainty to create impact: exploring the processes and transformative potential of co-produced research through development of a social impact model . Health research policy and systems. 2018 16(1):1-8.
25. Cowdell F. Knowledge mobilisation: an ethnographic study of the influence of lay mindlines on eczema self-management in primary care in the UK. BMJ open. 2018 Aug 1;8(8):e021498.
26. Cowdell F. Knowledge mobilisation: an ethnographic study of the influence of practitioner mindlines on atopic eczema self-management in primary care in the UK. BMJ open. 2019 Jul 1;9(7):e025220.
27. Cowdell F. Knowledge mobilisation: an exploratory qualitative interview study to confirm and envision modification of lay and practitioner eczema mindlines to improve consultation experiences and self-management in primary care in the UK. BMJ open. 2019 Jun 1;9(6):e028225.
28. Cowdell F, Ahmed T, Layfield C. Knowledge mobilisation: a UK co-creation study to devise strategies to amend lay and practitioner atopic eczema mindlines to improve consultation experiences and self-management practices in primary care. BMJ open. 2020 Sep 1;10(9):e036520.
29. Carlan NA, Kramer DM, Bigelow P, Wells R, Garritano E, Vi P. Digging into construction: Social networks and their potential impact on knowledge transfer. Work. 2012 Jan 1;42(2):223-32.
30. Thompson MR, Schwartz Barcott D. The role of the nurse scientist as a knowledge broker. Journal of Nursing Scholarship. 2019 Jan;51(1):26-39.
31. Nishida K, Dilworth DA. Fundamental Problems of Philosophy the World of Action and the Dialectical World. Translated with an Introd. By David A. Dilworth. 1970.
32. Nishida K. An Inquiry into the Good, as translated by Abe, M. and C. Ives.(1990). New Haven./London: Yale University, 1921.
33. Shimizu H. Ba-principle: new logic for the real-time emergence of information. Holonics 1995;5:67–79.

For peer review only - http://bmjopen.bmj.com/site/about/guidelines.xhtml

34. Nambozi, G (2014) An evaluation of community based university nursing education programme and stakeholders' experiences. https://hydra.hull.ac.uk/assets/hull:14394a/content (accessed 18 May 2021).
35. Newman ME. Ego-centered networks and the ripple effect. Social Networks. 2003 Jan 1;25(1):83-95.
36. Chin JH, Mansori S. Social marketing and public health: A literature review. Journal of Marketing Management and Consumer Behavior. 2018 May 20;2(2).
37. Evans WD, Christoffel KK, Necheles JW, Becker AB. Social marketing as a childhood obesity prevention strategy. Obesity. 2010 Feb 1;18(n1s):S23.
38. Bornbaum CC, Kornas K, Peirson L, Rosella LC. Exploring the function and effectiveness of knowledge brokers as facilitators of knowledge translation in health-related settings: a systematic review and thematic analysis. Implementation Science. 2015 10(1):1-2.
39. Marn TM, Wolgemuth JR. Applied Qualitative Data Analysis After the Ontological Turn. Qualitative Report. 2021 Jun 1;26(6).
40. Lather P, St. Pierre EA. Post-qualitative research. International journal of qualitative studies in education. 2013 Jul 1;26(6):629-33.
41. Mazzei LA, Jackson AY. Complicating voice in a refusal to "let participants speak for themselves". Qualitative inquiry. 2012 Nov;18(9):745-51.
42. Østern TP, Jusslin S, Nødtvedt Knudsen K, Maapalo P, Bjørkøy I. A performative paradigm for post-qualitative inquiry. Qualitative Research. 2021 Jul 7:14687941211027444.
43. National Institute for Health and Care Research. Involve Patients. Involve patients | NIHR (accessed 17 November 2022)
44. Tong A, Sainsbury P, Craig J. Consolidated criteria for reporting qualitative research (COREQ): a 32-item checklist for interviews and focus groups. *Int J Qual Health Care* 2007;19(6):349-357.
45. Labbé D, Mahmood A, Miller WC, Mortenson WB. Examining the Impact of Knowledge Mobilization Strategies to Inform Urban Stakeholders on Accessibility: A Mixed-Methods study. International journal of environmental research and public health. 2020 Jan;17(5):1561.
46. Davies HTO, Powell AE, Nutley SM. Mobilising knowledge to improve UK health care: learning from other countries and other sectors – a multimethod mapping study. NIHR Journals Library, Southampton (UK); 2015. PMID: 26110190.
47. Powell A, Davies H, Nutley S. Missing in action? The role of the knowledge mobilisation literature in developing knowledge mobilisation practices. Evidence & Policy: A Journal of Research, Debate and Practice. 2017 May 19;13(2):201-23.
48. Bayley JE, Phipps D, Batac M, Stevens E. Development of a framework for knowledge mobilisation and impact competencies. Evidence & Policy: A Journal of Research, Debate and Practice. 2018 Nov 26;14(4):725-38.
49. Abma TA, Cook T, Rämgård M, Kleba E, Harris J, Wallerstein N. Social impact of participatory health research: collaborative non-linear processes of knowledge mobilization. Educational action research. 2017 Aug 8;25(4):489-505.
50. Worton S, Loomis C, Pancer S, Nelson G, Peters RD. Evidence to impact: A community knowledge mobilisation evaluation framework. Gateways: International Journal of Community Research and Engagement. 2017 10:121-42.
51. Morton S, Wilson S, Inglis S, Ritchie K, Wales A. Developing a framework to evaluate knowledge into action interventions. BMC health services research. 2018 Dec;18(1):1-3.
52. Morton S. Progressing research impact assessment: A 'contributions' approach. Research Evaluation. 2015 Oct 1;24(4):405-19.

For peer review only - http://bmjopen.bmj.com/site/about/guidelines.xhtml

53. Nagtegaal R, Tummers L, Noordegraaf M, Bekkers V. Nudging healthcare professionals towards evidence-based medicine: a systematic scoping review. Journal of Behavioral Public Administration. 2019 Oct 2;2(2).
54. Khaleel I, Wimmer BC, Peterson GM, Zaidi ST, Roehrer E, Cummings E, Lee K. Health information overload among health consumers: a scoping review. Patient education and counseling. 2020 Jan 1;103(1):15-32
55. Teasdale E, Muller I, Sivyer K, Ghio D, Greenwell K, Wilczynska S, Roberts A, Ridd MJ, Francis N, Yardley L, Thomas KS. Views and experiences of managing eczema: systematic review and thematic synthesis of qualitative studies. British Journal of Dermatology. 2021 Apr;184(4):627-37.
56. Mueller SM, Hongler VN, Jungo P, Cajacob L, Schwegler S, Steveling EH, Thomas ZR, Fuchs O, Navarini A, Scherer K, Brandt O. Fiction, falsehoods, and few facts: cross-sectional study on the content-related quality of atopic eczema-related videos on YouTube. Journal of medical Internet research. 2020 Apr 24;22(4):e15599.
57. Slattery P, Saeri AK, Bragge P. Research co-design in health: a rapid overview of reviews. Health research policy and systems. 2020 Dec;18(1):1-3.
58. Gruffydd-Jones K, Hansen K. Working for better asthma control: how can we improve the dialogue between patients and healthcare professionals?. Advances in Therapy. 2020 Jan;37(1):1-9.
59. Scarlett J, Forsberg BC, Biermann O, Kuchenmüller T, El-Khatib Z. Indicators to evaluate organisational knowledge brokers: a scoping review. Health research policy and systems. 2020 Dec;18(1):1-3.
60. Ayatollahi H, Zeraatkar K. Factors influencing the success of knowledge management process in health care organisations: a literature review. Health Information & Libraries Journal. 2020 Jun;37(2):98-117.
61. Kislov R, Wilson P, Boaden R. The 'dark side'of knowledge brokering. Journal of health services research & policy. 2017 Apr;22(2):107-12.
62. Wye L, Cramer H, Carey J, Anthwal R, Rooney J, Robinson R, Beckett K, Farr M, le May A, Baxter H. Knowledge brokers or relationship brokers? The role of an embedded knowledge mobilisation team. Evidence & Policy: A Journal of Research, Debate and Practice. 2019 1;15(2):277-92.

For peer review only - http://bmjopen.bmj.com/site/about/guidelines.xhtml

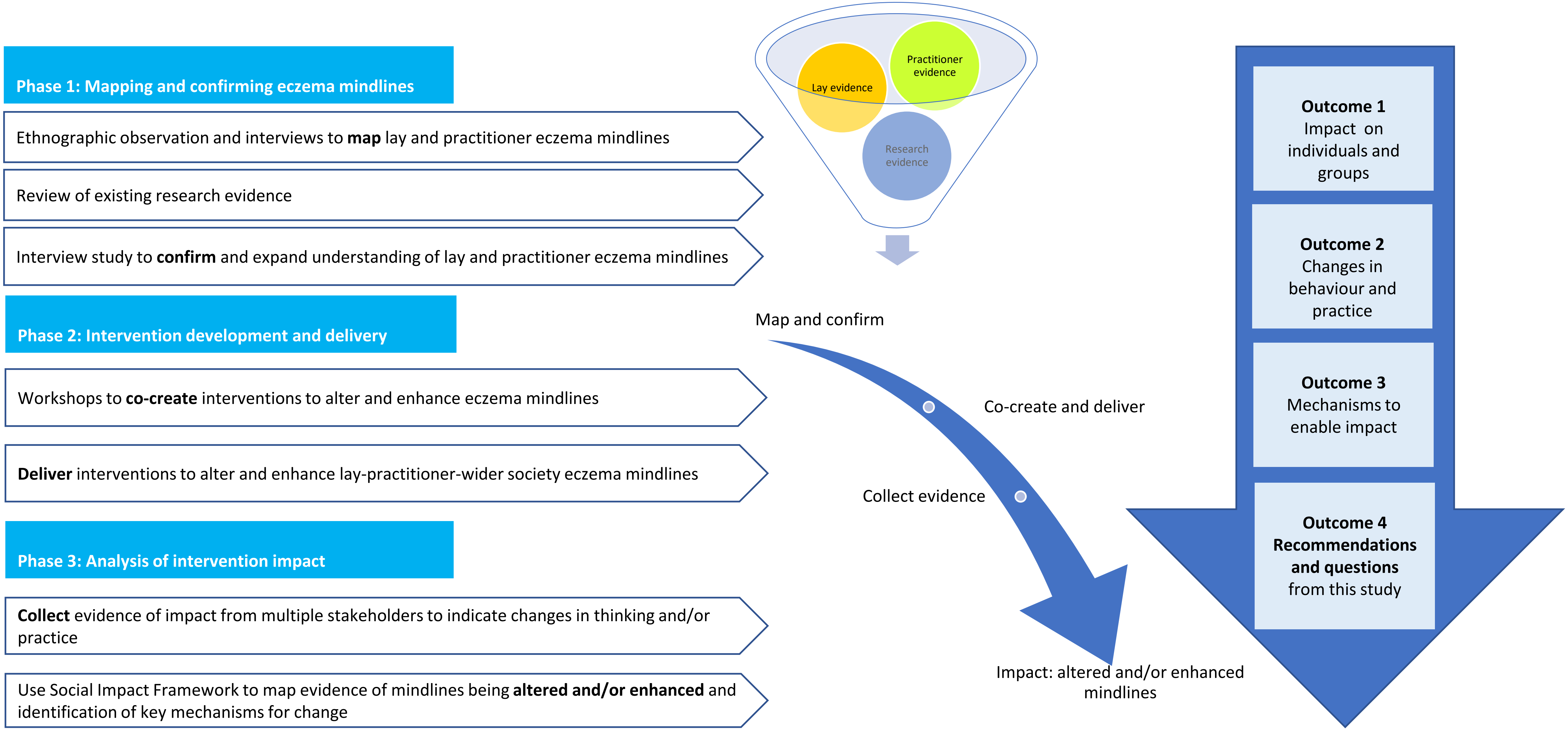
Phase 1: Mapping and confirming eczema mindlines
Ethnographic observation and interviews to map lay and practitioner eczema mindlines
Review of existing research evidence
Interview study to confirm and expand understanding of lay and practitioner eczema mindlines
Phase 2: Intervention development and delivery
Workshops to co-create interventions to alter and enhance eczema mindlines
Deliver interventions to alter and enhance lay-practitioner-wider society eczema mindlines
Phase 3: Analysis of intervention impact
Collect evidence of impact from multiple stakeholders to indicate changes in thinking and/or practice
Use Social Impact Framework to map evidence of mindlines being altered and/or enhanced and identification of key mechanisms for change
Lay evidence
Practitioner evidence
Research evidence
Map and confirm
Co-create and deliver
Collect evidence
Impact: altered and/or enhanced mindlines
Outcome 1
Impact on individuals and groups
Outcome 2
Changes in behaviour and practice
Outcome 3
Mechanisms to enable impact
Outcome 4
Recommendations and questions from this study

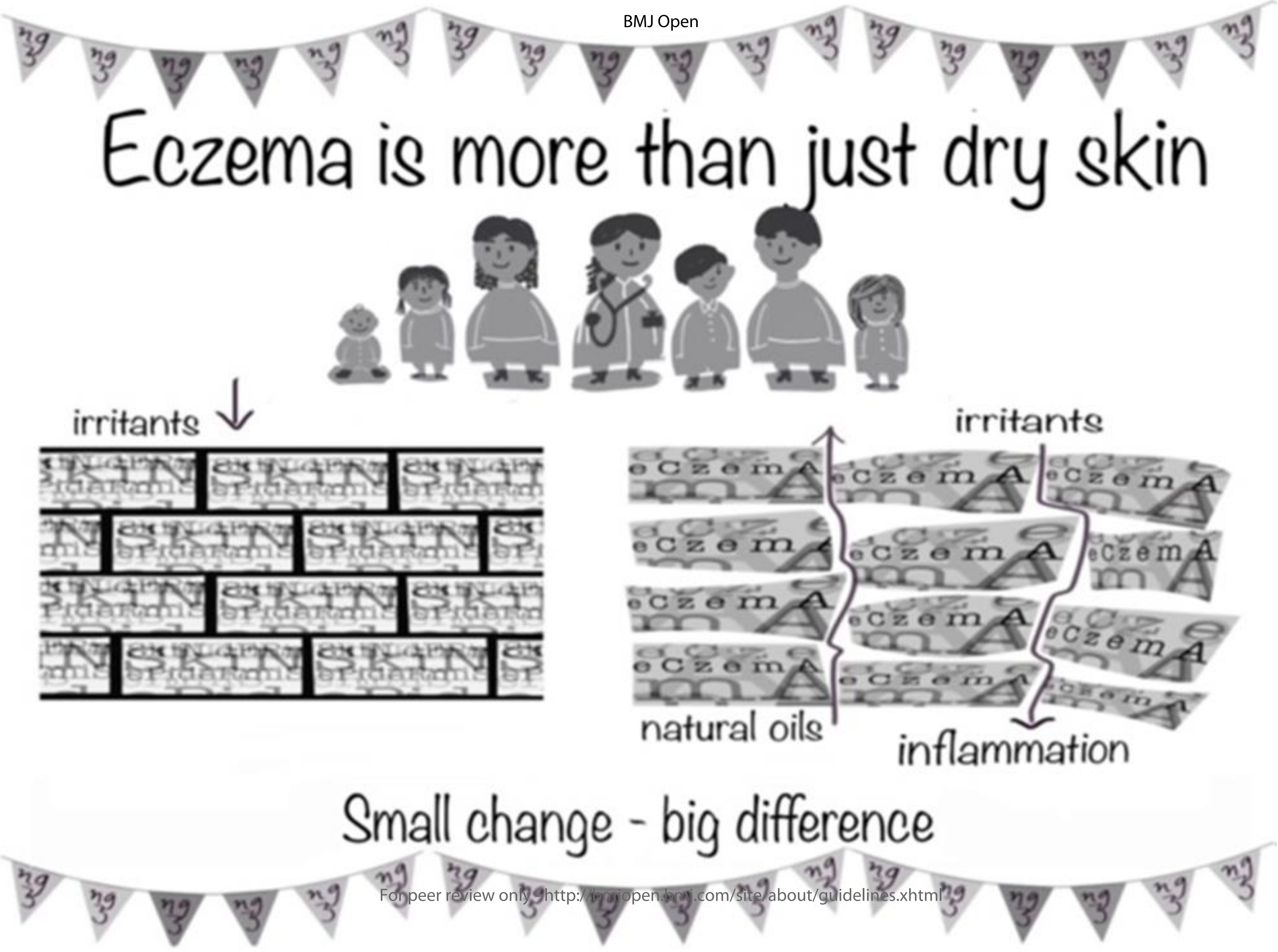
Eczema is more than just dry skin
irritants
irritants
natural oils
inflammation
Small change - big difference

BIRMINGHAM CITY University

ECZEMA IS MORE THAN JUST DRY SKIN

- 1 in 5 babies and children have eczema ('atopic eczema')
- Atopic eczema often runs in families
- There is no one cause
- A faulty skin barrier means the skin is dry, itchy and inflamed
- Eczema is made worse by 'triggers' which can be difficult to avoid

Information from Eczema Mindlines study, Birmingham City University

In association with

NATIONAL ECZEMA SOCIETY

FUNDED BY NIHR | National Institute for Health Research

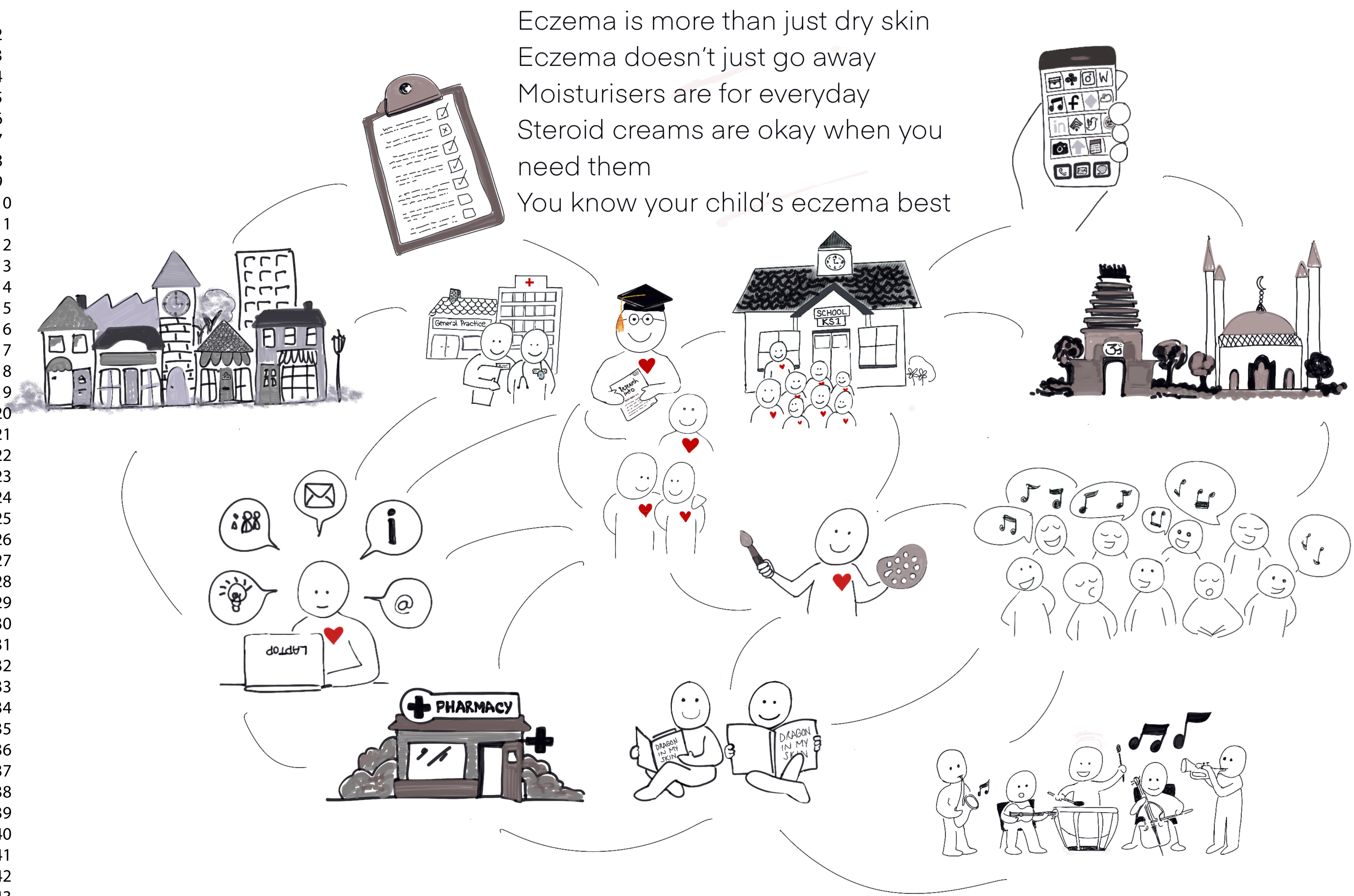
Eczema is more than just dry skin
Eczema doesn't just go away
Moisturisers are for everyday
Steroid creams are okay when you need them
You know your child's eczema best
General Practice
SCHOOL
KS1
PHARMACY
DRAGON IN MY SKIN
DRAGON IN MY SKIN

BMJ Open co-creation eczema mindlines COREQ 17.12.19

COREQ Statement

Knowledge mobilisation: An ethnographic study of the influence of practitioner mindlines on eczema self-management in primary care in the United Kingdom

Statement	Page no
**Domain 1: Research team and reflexivity**<br>*Personal Characteristics*<br>1. Interviewer/facilitator Which author/s conducted the interview or focus group?<br>FC was knowledge broker	5
2. Credentials What were the researcher's credentials? E.g. PhD, MD<br>DProf, RN	Title page
3. Occupation What was their occupation at the time of the study?<br>Professor of Nursing and Health Research	Title page
4. Gender Was the researcher male or female?<br>Female	Title page
5. Experience and training What experience or training did the researcher have?<br>I have extensive experience in qualitative research.	Title page
Relationship with participants<br>6. Relationship established Was a relationship established prior to study commencement?<br>**Knowledge broker**	Throughout manuscript
7. Participant knowledge of the interviewer<br>What did the participants know about the researcher? e.g. personal goals, reasons for doing the research	NA
8. Interviewer characteristics What characteristics were reported about the interviewer/facilitator? e.g. Bias, assumptions, reasons and interests in the research topic<br>Participants were aware, and it is reported in the manuscript, that I am a Registered Nurse with an interest in how eczema knowledge is developed and shared between patients and practitioners in primary care and that this was a publically funded study.	NA
**Domain 2: study design**<br>*Theoretical framework*<br>9. Methodological orientation and theory<br>What methodological orientation was stated to underpin the study? e.g. grounded theory, discourse analysis, ethnography, phenomenology, content analysis<br>Mindlines explained in background section	3-4
Participant selection<br>10. Sampling How were participants selected? e.g. purposive, convenience, consecutive, snowball<br>Purposive sampling to ensure a mix of co-creators	NA
11. Method of approach How were participants approached? e.g. face-to-face, telephone, mail, email	NA
12. Sample size How many participants were in the study?	NA
13. Non-participation How many people refused to participate or dropped out? Reasons?	NA
Setting<br>14. Setting of data collection Where was the data collected? e.g. home, clinic, workplace<br>Accessible conference centre and via email / telephone	8
15. Presence of non-participants Was anyone else present besides the participants and researchers?	NA
16. Description of sample What are the important characteristics of the sample? e.g. demographic data, date<br>Interview participants were sampled by profession, gender and years in practice.	Table 4
Data collection<br>17. Interview guide Were questions, prompts, guides provided by the authors? Was it pilot tested?	NA
18. Repeat interviews Were repeat interviews carried out? If yes, how many?	NA

For peer review only

BMJ Open co-creation eczema mindlines COREQ 17.12.19

19. Audio/visual recording Did the research use audio or visual recording to collect the data?	No
20. Field notes Were field notes made during and/or after the interview or focus group?<br>Notes and flip charts used	NA
21. Duration What was the duration of the interviews or focus group?	NA
22. Data saturation Was data saturation discussed?	NA
23. Transcripts returned Were transcripts returned to participants for comment and/or correction?<br>Data summaries were circulated to group members for comment, supplementation and modification	NA
**Domain 3: analysis and findings**<br>*Data analysis*<br>24. Number of data coders How many data coders coded the data?<br>Data analysis was completed by FC and RP	8
25. Description of the coding tree Did authors provide a description of the coding tree?<br>No	NA
26. Derivation of themes Were themes identified in advance or derived from the data?<br>Findings were derived from the data	8
27. Software What software, if applicable, was used to manage the data?<br>No	NA
28. Participant checking Did participants provide feedback on the findings?<br>Yes	NA
Reporting<br>29. Quotations presented Were participant quotations presented to illustrate the themes / findings? Was each quotation identified? e.g. participant number<br>Quotations are provided	8-15
30. Data and findings consistent Was there consistency between the data presented and the findings?<br>Yes.	8-15
31. Clarity of major themes Were major themes clearly presented in the findings?<br>Yes.	8-15
32. Clarity of minor themes Is there a description of diverse cases or discussion of minor themes?<br>Yes,	8-15

For peer review only
